# Ketogenic diet ameliorates axonal defects and promotes myelination in Pelizaeus–Merzbacher disease

**DOI:** 10.1007/s00401-019-01985-2

**Published:** 2019-03-27

**Authors:** Sina K. Stumpf, Stefan A. Berghoff, Andrea Trevisiol, Lena Spieth, Tim Düking, Lennart V. Schneider, Lennart Schlaphoff, Steffi Dreha-Kulaczewski, Annette Bley, Dinah Burfeind, Kathrin Kusch, Miso Mitkovski, Torben Ruhwedel, Philipp Guder, Heiko Röhse, Jonas Denecke, Jutta Gärtner, Wiebke Möbius, Klaus-Armin Nave, Gesine Saher

**Affiliations:** 1 Department of Neurogenetics, Max-Planck-Institute of Experimental Medicine, Hermann-Rein-Str. 3, 37075 Göttingen, Germany; 2 Division of Pediatric Neurology, Department of Pediatrics and Adolescent Medicine, University Medical Center, 37075 Göttingen, Germany; 3 University Children’s Hospital, University Medical Center Hamburg-Eppendorf, 20246 Hamburg, Germany; 4 Light Microscopy Facility, Max-Planck-Institute of Experimental Medicine, 37075 Göttingen, Germany; 5 Electron Microscopy Core Unit, Max-Planck-Institute of Experimental Medicine, 37075 Göttingen, Germany; 6 Center Nanoscale Microscopy and Molecular Physiology of the Brain (CNMPB), 37073 Göttingen, Germany

**Keywords:** Ketogenic diet, Myelin, Pelizaeus–Merzbacher disease, Remyelination, Axonal degeneration, Mitochondria

## Abstract

Pelizaeus–Merzbacher disease (PMD) is an untreatable and fatal leukodystrophy. In a model of PMD with perturbed blood–brain barrier integrity, cholesterol supplementation promotes myelin membrane growth. Here, we show that in contrast to the mouse model, dietary cholesterol in two PMD patients did not lead to a major advancement of hypomyelination, potentially because the intact blood–brain barrier precludes its entry into the CNS. We therefore turned to a PMD mouse model with preserved blood–brain barrier integrity and show that a high-fat/low-carbohydrate ketogenic diet restored oligodendrocyte integrity and increased CNS myelination. This dietary intervention also ameliorated axonal degeneration and normalized motor functions. Moreover, in a paradigm of adult remyelination, ketogenic diet facilitated repair and attenuated axon damage. We suggest that a therapy with lipids such as ketone bodies, that readily enter the brain, can circumvent the requirement of a disrupted blood–brain barrier in the treatment of myelin disease.

**Electronic supplementary material:**

The online version of this article (10.1007/s00401-019-01985-2) contains supplementary material, which is available to authorized users.

## Introduction

Primary defects in lipid metabolism are associated with myelin disease [13] and vice versa, a growing number of neurodegenerative diseases [1, 2, 14, 68], including the hereditary leukodystrophy Pelizaeus–Merzbacher disease (PMD), is associated with a perturbed brain lipid metabolism [29, 57, 63, 73]. In the nervous system, the majority of lipids is found in myelin sheaths, a multilayered stack of membranes synthesized by oligodendrocytes. Myelinating oligodendrocytes contribute to axon integrity by providing trophic support and electrical insulation for impulse propagation [23, 33]. Myelination failure is hence associated with axon damage and dysfunction leading to deficits in cognition and motor abilities.

PMD is most frequently caused by a duplication of the X-linked myelin gene *PLP1* (proteolipid protein 1) [11, 24, 40, 59, 74]. Pathological hypomyelination and symptoms such as nystagmus, hypotonia, spasticity, ataxia and retarded cognitive development are characteristic of PMD. At present, PMD lacks any therapeutic option and the symptomatic and supportive medications are directed at seizure or spasticity control. In PMD oligodendrocytes, overexpressed PLP accumulates together with cholesterol which impairs the intracellular transport of proteins and lipids to the growing myelin sheath [31, 57, 62]. In combination with the progressive loss of mutant oligodendrocytes, this leads to dysmyelination and demyelination in PMD [11, 53, 59]. The MRI pattern of diffuse hypomyelination in PMD caused by *PLP1* duplication is hence considered consequence of arrested brain maturation and lacks focal or inflammatory demyelination [9, 66, 71]. Despite variable disease severity of PMD duplication patients, hypomyelination improves only to a minor extent over time. At later stages, axon degeneration is also a feature of the PMD pathology, leading to cortical atrophy that likely contributes to neurological impairment [9, 35]. It has been shown in vitro and in vivo that lipid supplementation can enhance myelination in hypomyelinating pathologies and thereby promote repair [6, 21, 55–57]. However, under physiological conditions plasma cholesterol cannot cross the blood–brain barrier (BBB) [7]. In a previous study, we found that the BBB is perturbed in a line of *Plp1* transgenic mice (Plp1tg-72 [51]), a model of PMD, allowing the entry of cholesterol from the circulation into the CNS. In this model, dietary supplementation with cholesterol strongly improves myelination [57].

Here, we hypothesized that (1) the accessibility to the CNS determines the efficacy of lipid treatment, and (2) in the CNS, the different lipid classes target distinct aspects of the hypomyelinating pathology, i.e. the primary defect in myelin-forming oligodendrocytes and the subsequent damage of hypo/demyelinated axons. This study combines two important therapeutic targets, cholesterol for the support of remyelinating oligodendrocytes and ketone bodies for the metabolic support of the axonal compartment.

## Results and discussion

### Dietary cholesterol does not rescue hypomyelination in PMD patients

Active demyelination with inflammatory responses in patients with PMD [24] predicts at least minor BBB disturbances. Therefore, in an individual, compassionate use trial, we first asked whether we could translate the dietary treatment approach in two patients with PMD caused by *PLP1* duplication. Daily cholesterol supplementation of up to 590 mg/kg was well tolerated and blood lipid and glucose values remained in the normal range (Online Resource Supplemental Fig. 1). No adverse reactions were observed in the PMD patients, in agreement with cholesterol supplementation studies in patients with Smith-Lemli-Opitz disease [18]. However, a therapeutic benefit was not as obvious as in PMD mice. By MRI measurements, hypomyelination remained stable over 2 years of treatment (Online Resource Supplemental Fig. 2) which is consistent with the normal course of PMD disease [59, 66]. Taken together, dietary supplementation with high dose cholesterol was safe in our two PMD patients and long term disease monitoring will decide about the potential therapeutic benefit of this treatment strategy.

In Plp1tg-72 mice, the compromised BBB allowed access of cholesterol from the circulation into the brain [57]. In contrast, in PMD the comparative analysis of CSF and serum did not reveal gross disturbance of BBB integrity (Online Resource Supplemental Fig. 1a), in agreement with the lack of enhancing gadolinium signals in MRI in a PMD patient [43]. In a substrain of Plp1tg-72 mice with preserved BBB integrity (Online Resource Supplemental Fig. 3a-b), the dietary cholesterol supplementation only moderately ameliorated myelin disease (Online Resource Supplemental Fig. 3c-d), most likely reflecting the limited therapeutic success in PMD patients. Of note, PMD patients and both Plp1tg mouse strains show a comparable initial increase in serum cholesterol (Online Resource Supplemental Fig. 1b, Online Resource Supplemental Fig. 3e) [57] as expected from healthy adult individuals [30], suggesting that serum cholesterol alone does not suffice to predict treatment efficacy. Since the substrain of Plp1tg-72 mice more adequately models human PMD, we used this line, termed Plp1tgB below, in subsequent experiments.

### Ketogenic diet improves pathology in a PMD model with preserved BBB integrity

**Fig. 1:**
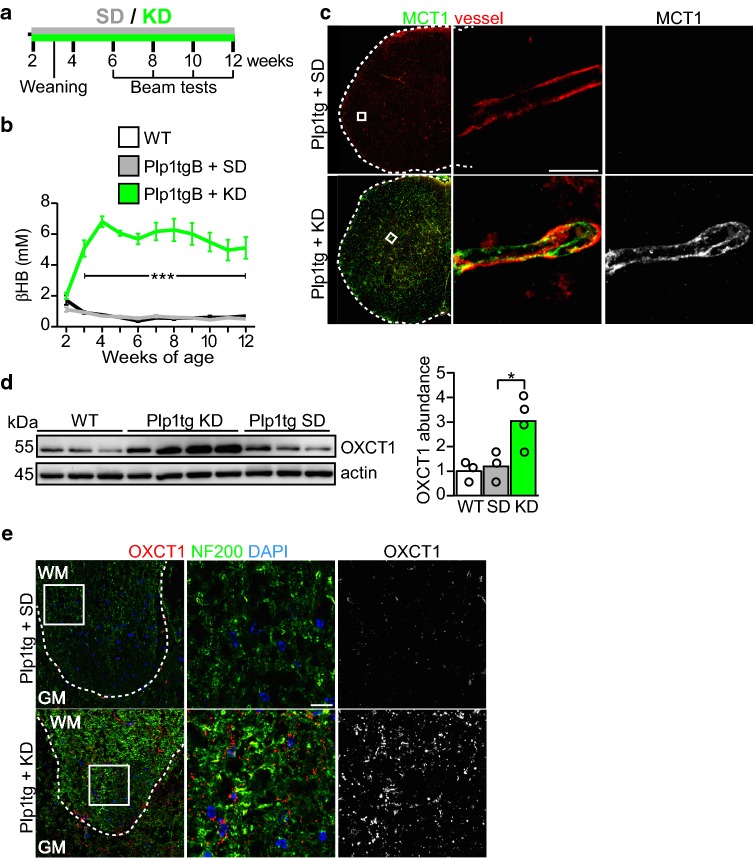
Increased ketone body uptake and ketolysis is induced in KD fed animals. **a** Treatment paradigm, Plp1tgB mice were fed ketogenic diet (KD) or standard chow (SD) between 2 and 12 weeks of age. **b** Mean blood levels of the ketone body beta-hydroxybutyrate (βHB) ± SEM. Significance was evaluated by 2way ANOVA with Tukey’s post test [*N *= 4 (wild type, WT), *N *= 8 (SD fed Plp1tgB), *N *= 9 (KD fed Plp1tgB)]. **c** Representative immunofluorescence detecting MCT1 on spinal cord sections of SD or KD fed Plp1tgB mice. Isolectin served to visualize blood vessels. The dashed line marks the tissue outline and the boxed area is enlarged on the right. **d** Western Blot with quantification of OXCT1 in wild type mice (*N *= 3), Plp1tgB mice fed SD (*N *= 3) or KD (*N *= 4). Equal protein loading was confirmed by staining of actin. Significance was tested using 1way ANOVA with Tukey’s multiple comparison test. **e** Immunolabeling of OXCT1 and neurofilament heavy chain (NF200) in the corticospinal tract of the spinal cord in Plp1tgB mice fed SD or KD. Dashed line marks the border between gray matter (GM) and white matter (WM) in dorsal spinal cord. The boxed area is enlarged on the right. Indicated are only significant differences between SD and KD fed Plp1tgB mice (*P < 0.05; ***P < 0.001). Scale bars 10 µm

The consumption of a high-fat/low-carbohydrate ketogenic diet causes the liver to generate ketone bodies. In the brain, ketone bodies such as beta-hydroxybutyrate facilitate sterol synthesis [32] which is essential for myelin membrane growth [55]. Thus, we asked whether a ketogenic diet that promotes CNS lipid metabolism under conditions of preserved BBB function [22, 32, 50] is also effective in hypomyelinating disease. Plp1tgB and control mice were given a ketogenic diet (KD) or standard a chow diet (SD) between 2 and 12 weeks of age and physiological parameters were monitored weekly (Fig. 1a). Already after 7 days of dietary intervention with KD, blood levels of ketone bodies strongly increased (about fivefold) and serum glucose dropped to about 70% of the level in SD fed mice (Fig. 1b, Online Resource Supplemental Fig. 4a, b). Ketone bodies are imported into the brain by monocarboxylate transporters, mainly MCT1 expressed by endothelial cells [37]. In accordance with ongoing ketosis, mRNA and protein levels of monocarboxylate transporters as well as enzymes of ketone body utilization significantly increased. This included the rate-limiting enzyme of the pathway, 3-oxoacid CoA-transferase 1 (OXCT1, also termed Succinyl-CoA:3-ketoacid coenzyme A transferase 1, SCOT, EC:2.8.3.5) whose abundance was threefold elevated in spinal cord of KD fed Plp1tgB mice, even more obvious by immunolabeling (Fig. 1d, e, Online Resource Supplemental Fig. 4c, d).

**Fig. 2:**
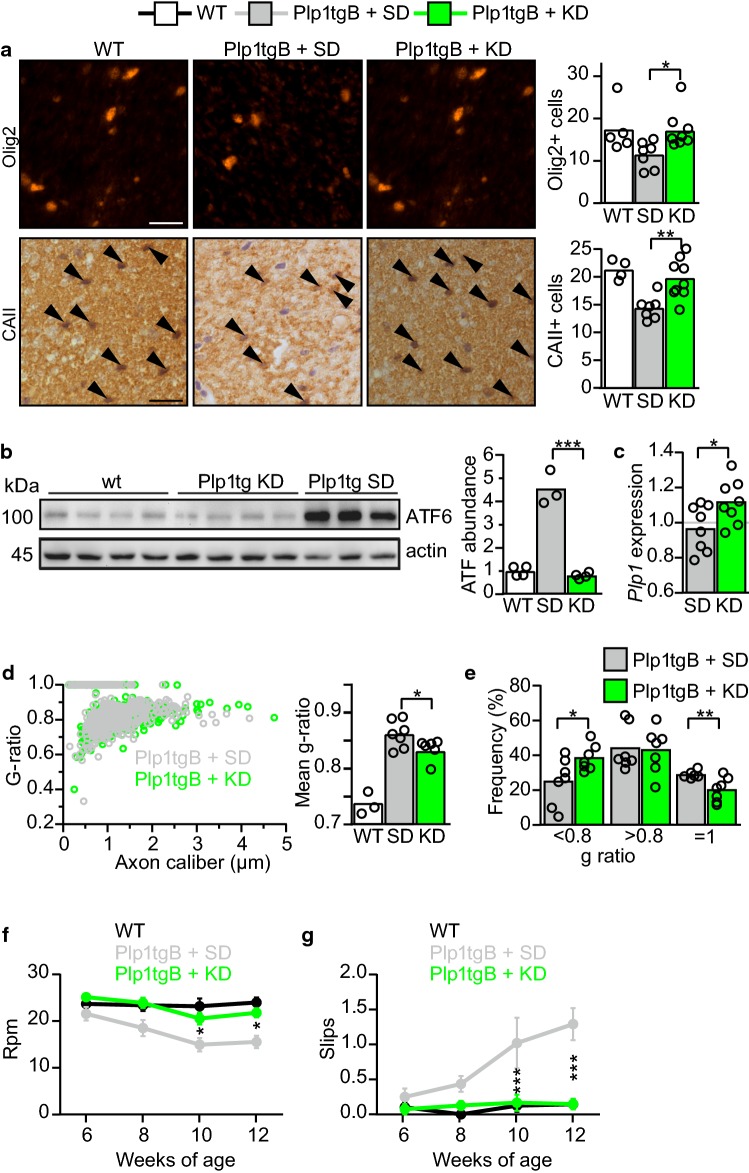
KD ameliorates PMD pathology in Plp1tgB animals. **a** Olig2 and CAII (arrowheads) immunolabeling of wild type and Plp1tgB mice fed SD and KD with quantification of cell numbers in dorsal white matter of the spinal cord on the right (*N *= 4–5 (WT), *N *= 7–8 (Plp1tgB fed SD), *N *= 8–9 (Plp1tgB fed KD), 1way ANOVA with Tukey’s post test). **b** Western Blot with quantification of ATF6 in lumbar spinal cord of wild type mice (*N *= 4), Plp1tgB mice fed SD (*N *= 3) or KD (*N *= 4). Equal protein loading was confirmed by reprobing for actin (1way ANOVA with Tukey’s post test). **c** Quantitative RT-PCR determining *Plp1* in spinal cord of Plp1tgB mice fed SD or KD (*N *= 8, 1way ANOVA with Sidak’s post test) normalized to wild type controls (*N *= 5, set to 1). **d** Quantification of myelination in the corticospinal tract from wild type mice, and Plp1tgB mice fed SD or KD (*N *= 7), showing *g*-ratio analysis as scatter plot (left panel) and the mean g ratio (right panel, 1way ANOVA with Tukey’s post test). **e** Relative frequency of sufficiently myelinated fibers (g ratio < 0.8), hypomyelinated fibers (*g* ratio > 0.8) or unmyelinated fibers (*g*-ratio = 1) in the CST of Plp1tgB fed SD or KD (*N *= 7, two-sided Student’s *t* test of each group). **f** Rotarod analysis and **g** elevated beam test performance at 6 to 12 weeks of age (*N *= 7–8; 2way ANOVA with Sidak’s post test). Indicated are only significant differences between SD and KD fed Plp1tgB mice (*P < 0.05; **P < 0.01; ***P < 0.001). Scale bars 20 µm

We next determined the degree of oligodendroglial defects, the hallmark of PMD pathology, in the corticospinal tract of the spinal cord from 12 weeks old Plp1tgB mice. As expected, the number of Olig2- and CAII-positive oligodendroglia was reduced in SD fed Plp1tgB mice, but feeding the KD diet rescued this defect in mutants (Fig. 2a). Moreover, untreated Plp1tgB mutant mice showed a robust endoplasmic reticulum stress response with about fourfold elevated ATF6 mRNA and protein levels. Both were significantly ameliorated in KD fed mutant mice (Fig. 2b, Online Resource Supplemental Fig. 4e). In agreement with the reduced oligodendroglial pathology, also astrogliosis and microgliosis were ameliorated in KD fed Plp1tgB compared to SD fed mutants (Online Resource Supplemental Fig. 5). *Plp1* mRNA overexpression is the primary defect in the classical form of PMD and in our mouse model [11, 51]. Feeding Plp1tgB mutants with KD did not lower *Plp1* expression but rather increased *Plp1* mRNA levels slightly (Fig. 2c), presumably reflecting enhanced oligodendrocyte survival. Indeed, the severe hypomyelination of untreated Plp1tgB mice was ameliorated by feeding a KD, as evidenced by fewer unmyelinated axons (*g*-ratio = 1) and more normally myelinated axons (*g*-ratio < 0.8), resulting in an overall reduction in mean *g*-ratio, as determined for the corticospinal tract (Fig. 2d, e). We next investigated whether feeding KD also ameliorated the clinical phenotype of PMD mice. When we assessed motor performance by two different behavioral tests, elevated beam test and rotarod test, untreated Plp1tgB mice showed progressive worsening of motor functions. By contrast, KD fed Plp1tgB animals retained their motor fitness at wild type levels (Fig. 2f, g). For comparison, feeding Plp1tgB mice a medium-chain triglyceride diet, which contains less fat than classical KD, elicited only a mild ketosis in our mice. This dietary regimen did not ameliorate motor performance, suggesting a critical role of ketone bodies rather than a direct effect of triglycerides/lipids (Online Resource Supplemental Fig. 6).

### Ketogenic diet ameliorates mitochondrial abnormalities in axons of PMD mice

**Fig. 3:**
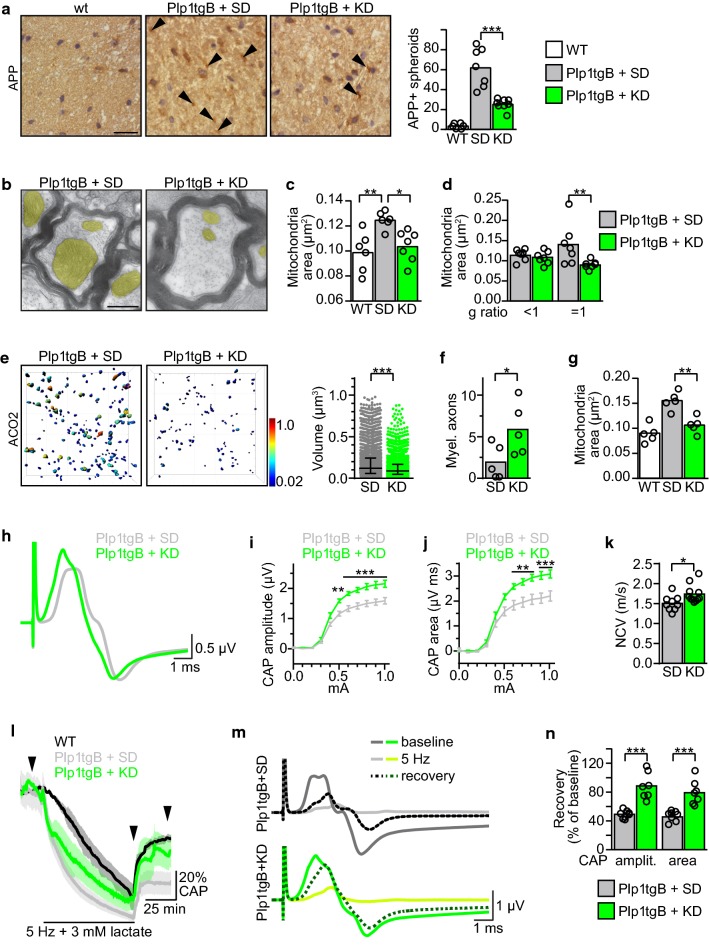
KD rescues mitochondria enlargement and ameliorates impulse conduction in Plp1tgB mice. **a** APP staining of axonal swellings/spheroids (arrowheads) in wild type and Plp1tgB mice fed SD and KD with quantification of cells per 30,000 µm^2^ corticospinal tract of the spinal cord (*N *= 6–8; 1way ANOVA with Tukey’s post test). Scale bar 20 µm. **b** Electron micrographs showing an example of enlarged axonal mitochondria (colored in yellow) in Plp1tgB mice fed SD and a normally sized mitochondrium in KD fed Plp1tgB mice. Scale bar 0.5 μm. **c** Mean area of axonal mitochondrial in cross-sectioned corticospinal tract of wild type mice, and Plp1tgB animals fed SD or KD (at least 60 mitochondria per animal were counted, *N *= 6–7 animals; 1way-ANOVA with Tukey’s post test). **d** Stratification of mitochondrial sizes shown in **c** with respect to myelinated axons (*g*-ratio < 1) and unmyelinated axons (*g*-ratio = 1) (two-sided Student’s *t* test of each group). **e** 3D modeling of ACO2 immunolabeled mitochondria in deconvolved confocal images from Plp1tgB mice fed SD or KD with quantification of mitochondrial volumes (SD, 3178 or KD, 2817 mitochondria; 4 animals per condition). Volume data are presented as median with interquartile range (Kolmogorov–Smirnov test). **f** Density of myelinated axons in the optic nerve of SD and KD fed Plp1tgB animals (mean per 100 square µm ± SEM of *N *= 4–5 animals counting 13 images per animal; two-sided Student’s *t* test). **g** Evaluation of mitochondrial area in axons of the optic nerve analogous to the analysis shown in **c** (*N *= 5 animals, 1way-ANOVA with Tukey’s post test). **h**–**n** Electrophysiological evaluation of (*N *= 9–11) optic nerves from SD or KD fed Plp1tgB mice, showing **h** mean CAP traces ± SEM, *I*-*V* curves of **i** the CAP amplitude and **j** the CAP area (2way ANOVA with Sidak’s post test), and **k** nerve conduction velocity (NCV, two-sided Student’s *t* test). **l** Mean CAP area ± SEM after challenging optic nerves with 5 Hz stimulation in the presence of 3 mM lactate for 75 min, followed by recovery in the presence of 3 mM lactate and 10 mM glucose (*N *= 7–8 optic nerves from Plp1tgB animals and 2 WT nerves). **m** Mean traces extracted from **l** (position marked by arrowheads) at baseline, at the end of the stimulation challenge, and after recovery. **n** Quantification of recovery showing mean CAP amplitude and CAP area with individual data points in relation to baseline (two-sided Student’s *t* test for each analysis) (*P < 0.05; **P < 0.01; ***P < 0.001)

It is unlikely that the moderately improved myelination explains the dramatic improvement in motor functions in KD fed Plp1tgB animals. In PMD patients, motor development is strongly retarded, and progressive axonal loss likely causes the gradual decline of motor functions already achieved [58]. In accordance, the frequent axonal swellings (spheroids) in Plp1tgB mice were strongly reduced when feeding a KD (Fig. 3a). In addition, we observed in Plp1tgB mice that many axons contained enlarged mitochondrial profiles (Fig. 3b), as observed before in other models of PMD [28, 45, 53]. Such morphological alterations can reflect increased activity and/or functional deficits in mitochondria which could both occur in PMD (see below).

By systematic quantification of electron microscopic images, we detected a significant about 20% area increase of mitochondrial profiles in axons from Plp1tgB animals compared to wild type controls. This enlargement was most evident in unmyelinated axons (Fig. 3c, d), and even detectable in young mutants (Online Resource Supplemental Fig. 7). Importantly, KD completely normalized this phenomenon, which was most evident in axons that remained unmyelinated, and correlated well with the reduced volume of mitochondria, modelled from deconvolved confocal image stacks (Fig. 3c–e, Online Resource Supplemental Fig. 8). We also determined mRNA and protein abundance of several mitochondrial markers in spinal cord lysates to correlate mitochondrial morphology to alterations in fusion or fission of mitochondria but neither of the tested markers showed significant differences between the two treatment groups of Plp1tgB animals, likely because this analysis reflect the sum of neuronal and glial mitochondria (Online Resource Supplemental Fig. 9). Notably, feeding a cholesterol supplemented chow to Plp1tg animals (Plp1tgB or Plp1tg-72) did not reverse the increased mitochondrial sizes, even where myelination was improved (Online Resource Supplemental Fig. 3f). This suggests that high-cholesterol and ketogenic diets supported distinct therapeutic pathways.

The latter prompted us to evaluate the impact of KD on axonal integrity in the absence of myelin and analyzed optic nerves of Plp1tgB animals. When untreated, this fiber tract is almost devoid of myelin at the age of 12 weeks but unmyelinated axons appear morphologically healthy and retinal ganglion cells are not degenerated [20, 57]. Here, the KD diet showed only a modest effect on hypomyelination (Fig. 3f), but ameliorated the enlarged axonal mitochondria, similar to our observations in the corticospinal tract (Fig. 3g).

Next, we assessed the functional significance of mitochondrial abnormalities in hypomyelinated axons by sensitive electrophysiological analyses. Specifically, in acute ex-vivo preparations of the optic nerve, we quantified nerve conduction and functional axon integrity, by comparing compound axon potentials (CAP) as described [54, 69]. As expected for hypomyelinated fibers [4, 54, 64], optic nerves of untreated Plp1tgB mice exhibited an abnormally attenuated evoked response with a strong after-hyperpolarization phase caused by elevated activity of potassium channels (Online Resource Supplemental Fig. 10). In KD fed Plp1tgB mutants, in contrast, CAP amplitude and CAP area was significantly increased compared to their SD fed littermates, implying that more axons contributed to evoked impulse propagation (Fig. 3h–j). Nerve conduction velocity of Plp1tgB mice was slightly increased in KD fed animals, likely reflecting the increased density of myelinated axons (Fig. 3f, k).

Glycolytic oligodendrocytes support the axonal energy metabolism by exporting lactate [23, 36]. To bypass this glial support and rather directly assess mitochondrial function in axons, we challenged the axonal energy metabolism during repeated firing. Specifically, we monitored CAPs in the optic nerve in the absence of glucose and in the presence of a suboptimal lactate concentration (3 mM) during 5 Hz stimulation for 75 min [10], followed by a recovery period in the presence of both lactate and glucose. In wild type optic nerves, this protocol led to a gradual reduction of CAPs to about 25% of baseline values (Fig. 3l), in agreement with recent findings in the corpus callosum [41]. In optic nerves from Plp1tgB mice, however, the resulting CAP decay was even stronger and more rapid than in wild type controls, demonstrating that the PMD pathology affects the ability of axons to utilize lactate to maintain conduction. Feeding a KD to Plp1tgB mice attenuated the CAP decay (of 5 Hz stimulated optic nerves ex vivo), suggesting that optic nerves from KD fed mice are more resilient to this metabolic stress (Fig. 3l, m). Moreover, in this protocol we noted a strong (80%) recovery of the original CAP amplitude in nerves from KD fed mice comparable to wild types, contrasting to only 40% recovery in SD fed mice (Fig. 3l–n). This finding demonstrates that PMD, which primarily perturbs myelination, also indirectly affects axonal energy metabolism. These data also suggest that axonal integrity can be uncoupled from the myelination status by the KD. That is, despite the marginally increased myelination of the optic nerve of Plp1tgB mice when fed the KD, dysmyelinated axons functionally improved, as evidenced by physiological recordings, and this effect was accompanied by morphological normalization of axonal mitochondria.

### Ketogenic diet promotes repair in a model of adult remyelination

**Fig. 4:**
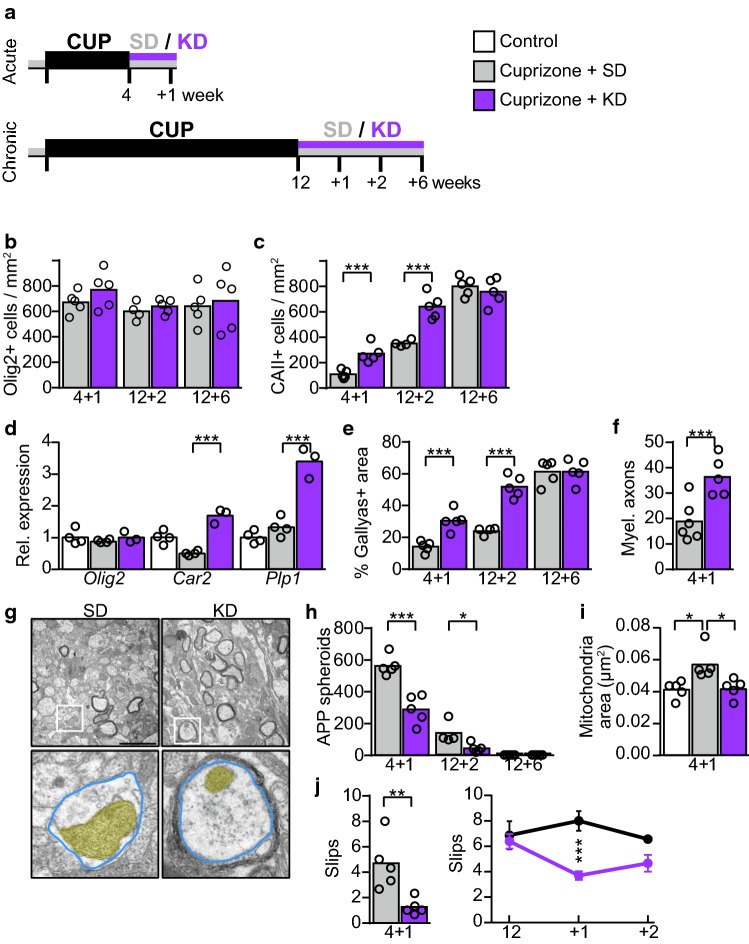
KD supports repair after cuprizone mediated demyelination. **a** Treatment paradigms. In an acute paradigm cuprizone (CUP) in SD was fed for 4 weeks, followed by feeding SD or KD for 1 week. In the chronic paradigm cuprizone was fed for 12 weeks, followed by KD or SD for up to 6 weeks. Histochemical quantification of **b** oligodendroglial cells (Olig2) and **c** mature oligodendrocytes (CAII) in the corpus callosum (two-sided Student‘s *t* test of each treatment cohort with *N *= 4–5 mice). **d** Relative gene expression of *Olig2*, *Car2* and *Plp1* in dissected corpus callosum of mice after 12 + 2 weeks paradigm, normalized to untreated controls (set to 1, *N *= 4). Significance was evaluated by 1way ANOVA with Tukey’s post test, indicated are only significant differences between cuprizone groups. **e** Myelin abundance (Gallyas silver impregnation, *N *= 4–5) and **f** number of myelinated axons per square 100 µm in the corpus callosum (*N *= 5–6). Data are expressed as mean with individual values (two-sided Student‘s *t* test of each treatment cohort). **g** Representative electron micrographs of the corpus callosum with boxed detail below (mitochondria colored in yellow, axonal outline colored in blue). Scale bar 2 µm. **h** Mean number of APP spheroids with individual values as a readout of axonal damage (two-sided Student‘s *t* test of each treatment cohort). **i** Mean size of axonal mitochondria in the corpus callosum (*N *= 5 animals, 1way ANOVA with Tukey’s post test). **j** Beam test to measure motor performance (*N *= 4–5), showing mean slips with individual data points (4 + 1, two-sided Student‘s *t* test) or mean ± SEM (12w-12 + 2 weeks, 2way ANOVA with Sidak’s post test) (*P < 0.05; **P < 0.01; ***P < 0.001)

Because of the therapeutic benefit of KD in the PMD mouse, a model of developmental hypomyelination, we compared in a 4-arm study the therapeutic potential of KD in a model of adult demyelination and remyelination. At the age of 8–10 weeks, wild type mice were demyelinated by feeding cuprizone, contained in a SD, for 4 weeks (acute paradigm) or for 12 weeks (chronic demyelination), followed by cuprizone withdrawal and remyelination with feeding SD or KD (Fig. 4a). Increased blood levels of the ketone body beta-hydroxybutyrate (βHB) and reduced blood glucose levels confirmed ketosis already at 7 days after the diet switch (Online Resource Supplemental Fig. 11a-d).

In agreement with our findings in Plp1tgB mice, mRNA expression levels of monocarboxylate transporters and enzymes of ketolysis were robustly increased in the corpus callosum of KD fed mice in comparison to SD fed controls and untreated wild type mice (Online Resource Supplemental Fig. 11e, f), likely reflecting ongoing ketone body utilization. Cuprizone leads to the death of most mature oligodendrocytes in the corpus callosum [39, 49], and after the withdrawal of cuprizone, myelin repair is reflected in an increasing number of oligodendrocyte lineage cells and gradual remyelination. While the number of Olig2 positive cells was not altered in KD fed mice compared to SD fed controls, the rate of oligodendrocyte precursor cell differentiation to CAII positive oligodendrocytes was strongly increased in KD fed mice (Fig. 4b–d, Online Resource Supplemental Fig. 11-12). In accordance, by feeding KD remyelination was accelerated about twofold at only 7 days after acute demyelination, and at 2 weeks after chronic demyelination (Fig. 4e–g).

In contrast, the degree of astrogliosis and microgliosis and expression of enzymes involved in detoxification of reactive oxygen species appeared unaffected by KD (Online Resource Supplemental Fig. 11 g-k, Online Resource Supplemental Fig. 12). Pro-inflammatory eicosanoids could contribute to pathogenesis in multiple sclerosis and expression of respective biosynthetic enzymes such as ALOX5 (arachidonate 5-lipoxygenase) was also increased in the chronic cuprizone model (Online Resource Supplemental Fig. 11 l) in accordance with previous reports [47, 75]. However, in contrast to a dietary intervention study with KD in MS patients [8], their expression was not altered by KD in our study. Axonal defects, as assessed by the density of APP positive axonal spheroids, was reduced by about 50% in KD fed mice compared to SD fed controls (Fig. 4h, Online Resource Supplemental Fig. 12). In addition, the cross-sectional size of axonal mitochondria, which were increased in SD fed mice during remyelination, even normalized (Fig. 4g, i). This suggests that the loss of oligodendroglial integrity rather than gliosis correlates with axonal perturbations. Finally, motor abilities, as quantified by an elevated beam test, also improved in KD fed mice (Fig. 4j). Thus, similar to our findings in developmental dysmyelination, KD also promoted functional repair in acute and chronic adult remyelination paradigms.

In this study, we have used two models of white matter disease with distinct etiologies and pathomechanisms, and found that feeding a KD is remarkably efficient in preserving axonal and oligodendroglial integrity. Plp1tgB mutants model the hereditary leukodystrophy PMD, in which overexpression of the myelin protein PLP induces cellular stress in differentiating oligodendroglia followed by dysmyelination and demyelination [67]. In contrast, the pharmacological cuprizone model induces transient demyelination and also assesses remyelination in adult mice [39, 49]. In both disease models, axon damage is likely secondary to the primary oligodendroglial defect [15, 34, 59], but a detailed understanding of the axonal injury is lacking. A combinatory effect of reduced metabolic support by injured oligodendrocytes and the exposure to pro-inflammatory factors, including reactive oxygen and nitrogen species, is most likely [15, 34]. In the PMD mouse model, we found mitochondrial enlargement not only in dysmyelinated axons but also in myelinated axons before overt demyelination, suggesting metabolic problems in axons at an early preclinical stage.

The KD dramatically changes the global metabolism and induces a plethora of alterations in the body including the CNS [50]. While discussed in many neurodegenerative diseases, KD is currently used in the management of epilepsy [3, 16, 70]. The mechanisms of seizure control are likely diverse, involve the gut microbiota [46] and may act in parallel, including effects on neurotransmission, epigenetic gene regulation, energy metabolism, and anti-inflammatory activity [16, 22, 61]. Our preclinical data reveal that KD provides metabolic support to axons in two distinct myelin pathologies that model key aspects of human myelin disease and mechanisms of recovery. We specifically note that also (chronically) unmyelinated axons appear to profit from KD.

Ketone bodies share some of the molecular targets with certain fatty acids that are contained in or processed from high-fat ketogenic diet regimens [3, 61]. However, in our study, a medium-chain triglyceride diet that only slightly raised blood ketone body levels was therapeutically ineffective, suggesting a critical and direct role of ketone bodies in the therapy of PMD.

We envision the following scenario of causal relations: (1) In PMD, PLP overexpression induces endoplasmic reticulum stress which affects oligodendrocytes and likely disturbs the oligodendroglial trophic support to axons. Chronic damage ultimately leads to loss of *Plp1*-overexpressing oligodendrocytes and demyelination. In the cuprizone model, pharmacologically induced oligodendrogliopathy causes demyelination. (2) To maintain impulse conduction unmyelinated axons require about five-fold more energy compared to the same tissue volume of myelinated axons [44]. Hence, aberrantly unmyelinated axons suffer from energy deficits because of the insufficient support by mutant oligodendrocytes and increased energy demands. (3) Energy deprived cells typically increase the activity of mitochondria. In addition, demyelination triggers inflammatory responses involving production of reactive oxygen and nitrogen species. Increased mitochondrial activity and oxidative stress are both associated with morphological changes and volume enlargement of mitochondria [38, 48] as observed in axons of both models of myelin disease. Chronic oxidative stress might damage mtDNA and mitochondrial enzymes [53] that could further trigger compensatory volume increases of mitochondria and aggravate degenerative processes. (4) In contrast to glucose, ketone bodies are directly metabolized by mitochondria fueling into the tricarboxylic cycle and oxidative phosphorylation. Since feeding a KD supports the conduction of optic nerve axons even in the absence of substantial myelination, we propose there is a direct supportive effect of KD on mitochondrial integrity in dysmyelinated axons. Bypassing the need for oligodendroglial trophic support could restore axonal mitochondria function (and morphology) and resolve energy deficits of axons. Relieving oligodendrocytes from the task to provide energetic support to axons might also contribute to the increased survival of mutant oligodendrocytes and to the ameliorated PMD pathology.

In contrast to the developmental PMD model, adult remyelination in the cuprizone model strongly benefitted from the KD, in line with previous findings using a fasting mimicking diet [12]. Here, we suggest that a compromised BBB in cuprizone fed animals allows for the entry of circulating cholesterol into the CNS (further enhanced by the KD that contains 0.1% cholesterol) [5, 6]. While the BBB may remain largely intact in classical human PMD, it is perturbed in other myelin diseases with a stronger inflammatory component. We conclude that there is a clear rationale to consider KD or a derivative as a future therapy for myelin diseases as it combines two important therapeutic targets, cholesterol for the support of remyelinating oligodendrocytes and ketone bodies for the metabolic support of the axonal compartment, and future clinical trials will reveal its feasibility in the management of demyelinating episodes.

## Materials and methods

### Patients

Two patients diagnosed Pelizaeus–Merzbacher disease (PMD) with hemizygote duplication of the complete *PLP1* gene were treated with high-dose oral cholesterol, conducted in two different clinical centers. Patient PMD-A was 29 months at start of dietary cholesterol supplementation. The final dose of 200 mg/kg/day was reached within 2 weeks. Cardiologic assessments including ECG, echocardiography and Doppler sonography were performed on annual basis and revealed normal results. Patient PMD-B was 30 months at start of dietary cholesterol supplementation. Over a period of 17 months the initial dose of 125 mg/kg/day was increased to 590 mg/kg/day and maintained for now further 13 months. Carotid artery intima-media thickness was determined to detect possible vascular side-effects of the cholesterol treatment which did not occur. Serum lipid levels of both patients were monitored regularly and remained in normal range [17]. Repeated measurements of blood and CSF glucose levels yielded normal results. CRP values stayed within the normal range. Evaluation of the developmental and clinical disease status [72] included general physical parameters, neurologic and developmental scores (ET6-6; WPPSI, Wechsler Preschool and Primary Scale of Intelligence), motor function (GMFCS, gross motor function classification system), as well as neurophysiologic and ophthalmologic assessments. Reiber analysis of albumin concentrations Q_alb_ ([albumin]_CSF_/[albumin]_serum_) served to detect gross blood–brain barrier abnormalities [52] which did not occur. The control subject was diagnosed with neurological asymptomatic adrenoleukodystrohy who undergo regular cranial MRIs as surveillance for onset of inflammatory demyelination.

### Magnetic resonance imaging (MRI)

Cranial MRI studies conducted at a 1.5T (Ingenia, Philips) (Hamburg) or a 3T clinical scanner (Tim TRIO, Siemens Healthcare) (Göttingen) comprised conventional T1- and T2-weighted images. In addition, semiquantitative magnetization transfer (MT) imaging was performed using a 3D FLASH (fast low angle shot) sequence with 1.25 mm isotropic resolution and 240 mm field-of-view. MT contrast was imposed upon a proton density weighted reference (TR/TE/*α* = 25/4.9 ms/5°, 3.5 min measurement using partial acquisition techniques) by applying a 12.8 ms Gaussian MT-pulse of 540° nominal flip angle 2.2 kHz off resonance prior to excitation. By means of a second T1-weighted reference (TR/*α* = 11 ms/15°, 1.5 min), maps of the percentage MT saturation (MTsat maps) were calculated as described [19, 25–27]. The blue-gray-red-yellow color scale of the MTsat maps covered a range from − 0.1 pu (light blue; lower limit of the CSF mode) to 1.2 pu (gray, GM) to 2.5 pu (yellow; WM). The upper threshold was chosen post hoc below the MTsat values of myelinated WM of controls (3 pu) to increase the sensitivity in the range observed with severe hypomyelination.

### Mice

*Plp1* transgenic Plp1tg-72 [51] mice harbor three copies of the murine *Plp1* gene. The substrain of Plp1tg-72 mice (Plp1tgB) spontaneously developed after infection with helminthes and worms followed by standard treatment. While severity of histopathology differs between Plp1tg mouse strains, *Plp1* overexpression in spinal cord remained unchanged at about 1.2 fold normalized to wild type mice (Online Resource Supplemental Fig. 13). Wild type C57BL6 mice were obtained from Charles River. Only male mice were used in this study. Animals were allocated to cages of 4 animals per cage by block randomization for adaptation to the new environment for 2 weeks before cages were assigned to the different treatment groups.

#### Diets

Mice were fed normal chow (ssniff V1124 containing 11% fat, 36% protein, 53% carbohydrates (5% sugar) metabolizable energy), high-fat/low-carbohydrate ketogenic diet (ssniff E15149-30 containing 94% fat, 5% protein, 1% carbohydrates/sugar), medium-chain triglyceride diet (ssniff modified Surwit diet containing 70% fat, 15% protein, 15% carbohydrates (1% sugar)) or a normal chow supplemented with 0.5–5% w/w cholesterol.

#### Cuprizone

Adult male C57BL/6N mice (8–10 weeks of age) were randomly assigned to an experimental group. Mice were fed 0.2% w/w cuprizone (bis-cyclohexanone oxaldihydrazone, Sigma-Aldrich) in powdered chow (ssniff V1120) for 4 weeks (acute paradigm) or 12 weeks (chronic paradigm), followed by cuprizone withdrawal and feeding mice SD or KD ad libitum.

#### Motor coordination

For the elevated beam test, mice were put on a beam (width 1.5 cm) and allowed to run toward a hiding box. The number of slips in a defined 55 cm distance was assessed as a mean of two repeats per time point. For rotarod analysis, mice were placed on the rotarod starting with a speed of 4 rpm. Speed was increased with 10 rpm/min until 26 rpm was reached and held for 10 s. Maximum trial duration was 150 s and the latency to fall was recorded.

### Antibodies

The following antibodies were used: actin (Sigma A3853), ACO2 (Sigma, HPA001097), APP (Chemicon MAB348), ATF6 (Abcam 40256), CAII (Said Ghandour), GFAP (Chemicon MAB3402), Iba1 (Wako 019-19741), MAC3 (Pharmigen 01781D), NF200 (Sigma N4142), Olig2 (Charles Stiles/John Alberta, DF308), OXCT1 (Proteintech Europe 12175-1AP), SMI31 (Covance SMI-31P), VDAC (Rockland), isolectin IB4 coupled to Alexa 594 (Vectorlab). For generation of MCT1 (SLC16a1) antisera, rabbits were immunized with the intracellular peptide 221–236 of mouse MCT1 (CDANTDLIGGSPKGEKL). Anti-MCT1 antibodies were purified by affinity chromatography.

### Histochemistry

Mice were sacrificed by cervical dislocation and immersion fixed for 48 h or perfused with 4% formaldehyde (PFA). Brain samples of cuprizone treated animals were cut at Bregma 1.58 for comparable pathology. Tissue embedded in paraffin and cut into 5 µm sections (HMP 110, MICROM). Gallyas silver impregnation was done as described [6]. For immunohistological analyses, sections were deparaffinized followed by antigen-retrieval in sodium citrate buffer (0.01 M, pH 6.0). For immunofluorescence, sections were blocked with serum free protein block (Dako). Primary antibodies were diluted in 2% bovine serum albumin (BSA)/PBS and incubated for 48 h followed by fluorophor coupled secondary antibodies.

For volume analysis of mitochondria, oversampled images suitable for subsequent deconvolution were acquired with a Zeiss LSM 510 Meta confocal laser-scanning microscope (CLSM). A 63× (NA = 1.25) oil immersion objective was used to record images composed of 48 nm pixels, with *z*-planes being acquired every 190 nm. Signal-to-noise, resolution signal and sample geometry of the resulting *z*-stacks were corrected by deconvolution using the Huygens Professional software package (version 18.04, Scientific Volume imaging B.V., Hilversum, Netherlands). An image stack volume of 49.05 × 49.05 × 5.5 µm was used to generate 3D models of mitochondria by the Imaris software package (version 9.2.1, Bitplane AG, Zurich, Switzerland) of which the volumetric information in the range of 0.02–1 µm^3^ was extracted.

For immunohistochemistry, endogenous peroxidase activity was blocked with 3% hydrogen peroxide. Sections were then blocked (20% goat serum in BSA/PBS) and incubated with primary antibodies. Detection was done with the LSAB2 kit (Dako, Hamburg, Germany) or the Vector Elite ABC kit (Vector Labs). HRP substrate 3,3′-Diaminobenzidine was applied by using the DAB Zytomed Kit (Zytomed Systems GmbH). Haematoxylin stain was done to label nuclei. Sections were dehydrated prior to mounting (Eukitt). Specimens were analyzed on an Axio Imager.Z1 (Zeiss) equipped with an AxioCam MRc3, 0.63 × Camera Adapter and the ZEN 2012 blue edition software using 10× objective (Plan Apochromat 10×/0.45 M27) or 20× objective (Plan-Apochromat 20×/0.8) and evaluated with Image J software. Quantification of areas (Gallyas, GFAP, MAC3) were done by applying semi-automated Fiji software [60] macro to threshold (variable threshold in case of Gallyas and fixed threshold for antibody staining) and color deconvolute the images of the spinal cord or corpus callosum above the fornix. Three to five sections per animal were analyzed for quantification.

Electron microscopic analysis was done as previously described [6, 57]. Briefly, tissue was fixed in 4% PFA, 2.5% glutaraldehyde, 0.1 M phosphate buffer. Sagittal brain sections were cut on a vibratome (Leica VT1200, 300 µm) and the corpus callosum with adjacent tissue (-0.04 mm lateral) was punched with a 2 mm diameter punching tool. Tissue punches or spinal cord sections were embedded in epon (LynxII, EMS). At least 15 digital pictures using an on-axis 2048 × 2048-CCD camera (12,000 × magnification, TRS, Moorenweis) of uranyl acetate contrasted ultrathin sections were taken with the LEO912 electron microscope (Zeiss, Oberkochen). For quantification of mitochondrial sizes, the area at least 60 mitochondrial profiles per animal were measured. Only mitochondria of cross sectioned axons were considered. For electron microscopic analysis on high pressure frozen samples, optic nerves of P23 old mice were freshly prepared and cryo-immobilized by high pressure freezing using a HPM100 instrument (Leica, Vienna) and 20% PVP in PBS as filler. Freeze substitution was carried out as described [42].

### Protein analysis

Tissue samples were lysed in sucrose buffer (18% sucrose, 10 mM Tris/HCl pH 7.4, 1 mM sodium bicarbonate, 1 mM magnesium chloride, 0.1% Triton, 0.2% lithiumdodecyl sulphate, 0.025% sodium deoxycholate) with protease inhibition (Roche) using a Precellys 24 homogenizer (Bertin technologies). SDS gel electrophoresis, semi-dry blotting on PVDF membranes (Hybond P, Biosciences) and antibody incubations were done using standard procedures. Detection of immunolabeled proteins was performed with Western-Lighting Plus-ECL Reagent (Perkin Elmer) using ChemoCam Imager (Intas).

### Expression analysis

Expression analyses were done as described [6]. RNA was extracted from dissected tissue using QIAshredder and RNeasy protocols (Qiagen). Concentration and quality of RNA was evaluated using a NanoDrop spectrophotometer and RNA Nano (Agilent). cDNA was synthesized with Superscript III (Invitrogen) and quantitative PCRs were done in triplicates with the GoTaq qPCR Master Mix (Promega) on a 7500 Fast Real-Time PCR System (Applied Biosystems). Expression values were normalized to the mean of two housekeeping genes, *Hprt* (hypoxanthin-phosphoribosyl-transferase 1) and *Rplp0* (60S acidic ribosomal protein P). Quantification was done by applying the ΔΔCt method, normalized to age matched untreated controls (set to 1). All primers were intron-spanning. Expression of the following genes was measured: *Acat1* (acetyl-Coenzyme A acetyltransferase 1), *Aif1* (allograft inflammatory factor 1), *Alox5* (arachidonate 5-lipoxygenase), *Atf4* (activating transcription factor 4), *Atf6*, *Bdh1* (3-hydroxybutyrate dehydrogenase 1), *Hspa5* (heat shock protein 5) also termed *Bip* or *Grp78*, *Car2* (carbonic anhydrase 2), *Cat* (catalase), *Cox1* (cyclooxygenase 1), *Ddit3* (DNA-damage inducible transcript 3) also termed *Chop* (C/EBP homoologous protein), *Dmn1* *l* (dynamin 1 like), *Gfap* (glial fibrillary acidic protein), *Slc16a1* (solute carrier family 16 member 1, also termed monocarboxylate transporter 1 MCT1), *Mct2/Slc16a7*, *Olig2* (oligodendrocyte lineage transcription factor 2), *Pdgfa* (platelet derived growth factor alpha), *Ppargc1a* (peroxisome proliferative activated receptor, gamma, coactivator 1 alpha), also termed *Pgc1a* (PPAR gamma coactivator-1), *Plp1* (proteolipid protein 1), *Sod1* (superoxide dismutase 1), *Sod2*, *Tfam* (transcription factor A, mitochondrial), *Vdac* (voltage dependent anion channel 1), *Xbp1* (X-box binding protein 1). All primer sequences are listed in Table S1.

### Electrophysiological recordings

Electrophysiological recordings were done as described [54, 69]. Optic nerves were excised from decapitated mice, placed into an interface perfusion chamber (Harvard Apparatus, Holliston, MA) and continuously superfused with artificial cerebrospinal fluid (aCSF). The perfusion chamber was continuously aerated by a humidified gas mixture of 95% O_2_/5% CO_2_ and experiments were performed at 37 °C. Custom-made suction electrodes back-filled with aCSF were used for stimulation and recording as described [54, 65]. The stimulating electrode, connected to a battery (Stimulus Isolator 385; WPI, Berlin, Germany) delivered a supramaximal stimulus of 0.75 mA to the nerve evoking compound action potentials (CAP). The recording electrode was connected to an EPC9 amplifier (Heka Elektronik, Lambrecht/Pfalz, Germany). The signal was amplified 500 times, filtered at 30 kHz, and acquired at 100 kHz. The CAP, elicited by the maximum stimulation of 0.75 mA, was recorded at baseline stimulation frequency at 0.1 Hz in the presence of 10 mM glucose and 3 mM lactate with or without bath application of 50 µM 4-aminopyridine (4-AP). To determine the energetic capability of axons to sustain repeated firing, we depleted optic nerves of endogenous energy stores for 5 min (no substrate) followed by incubation in 3 mM lactate in the presence of continuous 5 Hz stimulation which was applied consisting of 100 stimuli, separated by 460 ms, during which the CAP was recorded. After 75 min, stimulation settings were switched back to baseline conditions and the CAP recovery was assessed. For *I*–*V* measurements, the evoked response of the optic nerve was elicited every 10 s, by subsequent 0.1 mA stimulation intensity steps, ranging from 0 to 1 mA.

### Tracer studies

Bodipy-cholesterol (Topflour, Avanti Polar Lipids) was injected i.p. (5 µg/g body weight). After 1 week, mice were perfused with PBS to remove tracer from the circulatory system. Evans Blue (50 mg/g body weight) was i.v. injected and mice were flushed after 4 h incubation as described [5]. Brains were dissected and immediately frozen on dry ice, weighed and stored at − 80 °C for further processing. Tissue was lyophilized (Christ LMC-1 BETA 1-16) at −36 °C for 24 h under vacuum of 0.2 mBar. For tracer extraction, hemispheres were incubated shaking in 10 µl formamide per mg brain at 57 °C for 24 h. Integrated density of tracer fluorescence was determined in triplicates on a fluorescent microscope (Observer Z2, Zeiss, Germany), equipped with an AxioCam MRc3, 1× Camera Adapter and the ZEN 2012 blue edition software recorded at 10× magnification (Plan-Apochromat 10×/0.45 M27). Tracer concentration was calculated using a standard curve and normalized to matched controls (set to 1).

### Statistical analysis

Statistical evaluation was done using GraphPad Prism (GraphPad Inc.), applying either unpaired two-sided Student’s t test for pairwise comparisons, ANOVA for comparisons of more than two groups or the Kolmogorov–Smirnov test for non-parametric comparisons, as stated in the figure legends. ANOVA was combined with a post test to evaluate individual groups. For all statistical tests, significance was measured against an alpha value of 0.05. Sample sizes are given in the figure legends. All error bars show SEM. *P* values are shown as *P < 0.05; **P < 0.01; ***P < 0.001. No statistical methods were used to predetermine sample sizes, but our sample sizes are similar to those reported in previous publications [6, 57]. Data analysis was performed blind to the experimental groups.

## Electronic supplementary material

Below is the link to the electronic supplementary material.

Supplementary material 1 (PDF 3826 kb)

Supplementary material 2 (DOCX 18 kb)
