# Correction to: Ketogenic diet ameliorates axonal defects and promotes myelination in Pelizaeus–Merzbacher disease

**DOI:** 10.1007/s00401-019-02064-2

**Published:** 2019-09-03

**Authors:** Sina K. Stumpf, Stefan A. Berghoff, Andrea Trevisiol, Lena Spieth, Tim Düking, Lennart V. Schneider, Lennart Schlaphoff, Steffi Dreha-Kulaczewski, Annette Bley, Dinah Burfeind, Kathrin Kusch, Miso Mitkovski, Torben Ruhwedel, Philipp Guder, Heiko Röhse, Jonas Denecke, Jutta Gärtner, Wiebke Möbius, Klaus-Armin Nave, Gesine Saher

**Affiliations:** 1 Department of Neurogenetics, Max-Planck-Institute of Experimental Medicine, Hermann-Rein-Str. 3, 37075 Göttingen, Germany; 2 Division of Pediatric Neurology, Department of Pediatrics and Adolescent Medicine, University Medical Center, 37075 Göttingen, Germany; 3 University Children’s Hospital, University Medical Center Hamburg-Eppendorf, 20246 Hamburg, Germany; 4 Light Microscopy Facility, Max-Planck-Institute of Experimental Medicine, 37075 Göttingen, Germany; 5 Electron Microscopy Core Unit, Max-Planck-Institute of Experimental Medicine, 37075 Göttingen, Germany; 6 Center Nanoscale Microscopy and Molecular Physiology of the Brain (CNMPB), 37073 Göttingen, Germany

## Correction to: Acta Neuropathologica (2019) 138:147–161 10.1007/s00401-019-01985-2

The original article was published with an erroneously duplicated image in Fig. 2a. The corrected Fig. 2a is given in the next page.

**Fig. 2:**
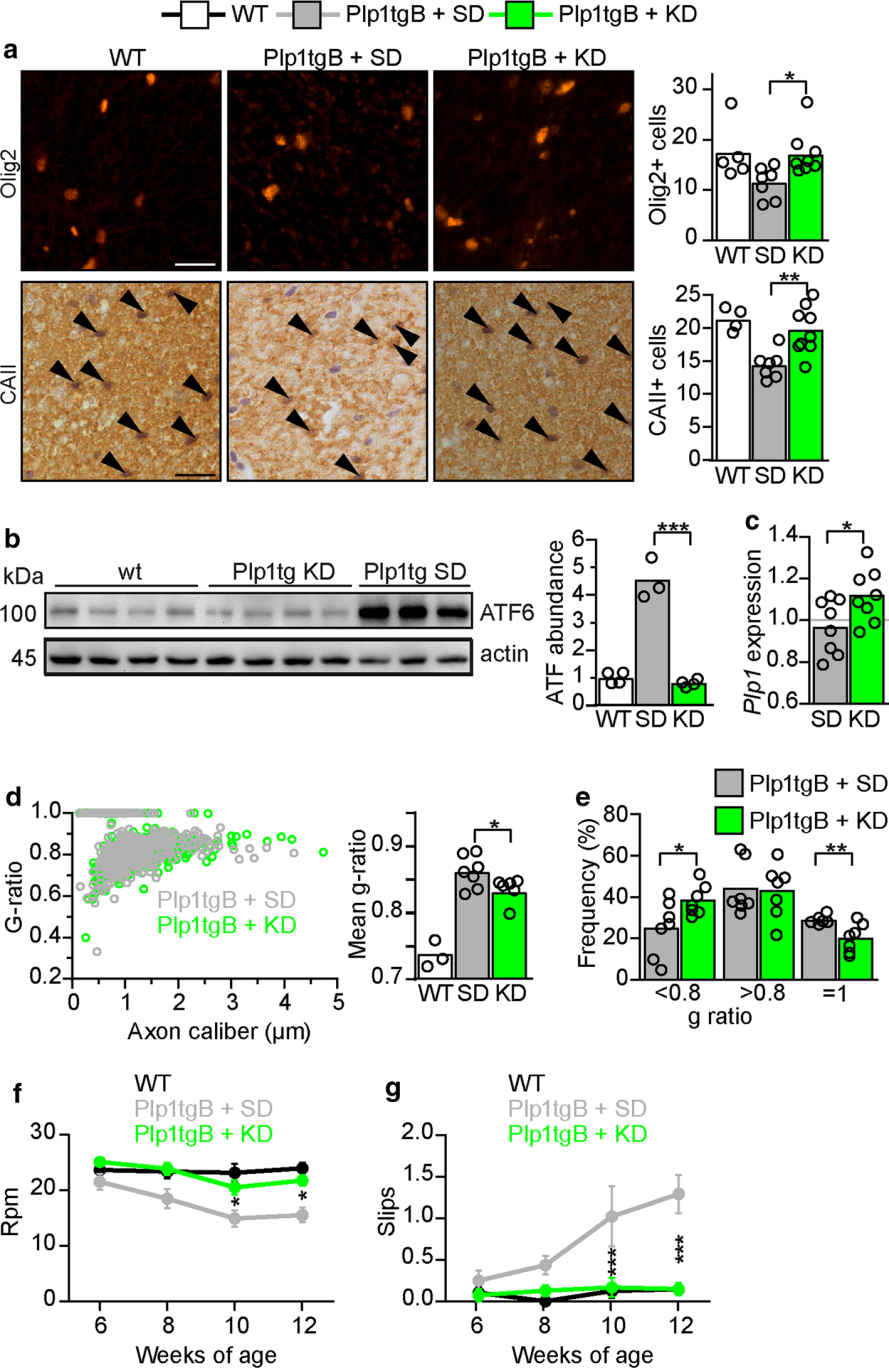
KD ameliorates PMD pathology in Plp1tgB animals. **a** Olig2 and CAII (arrowheads) immunolabeling of wild type and Plp1tgB mice fed SD and KD with quantification of cell numbers in dorsal white matter of the spinal cord on the right (*N* = 4–5 (WT), *N* = 7–8 (Plp1tgB fed SD), *N* = 8–9 (Plp1tgB fed KD), 1way ANOVA with Tukey’s post test). **b** Western Blot with quantification of ATF6 in lumbar spinal cord of in wild type mice (*N* = 4), Plp1tgB mice fed SD (*N* = 3) or KD (*N* = 4). Equal protein loading was confirmed by reprobing for actin (1way ANOVA with Tukey’s post test). **c** Quantitative RT-PCR determining Plp1 in spinal cord of Plp1tgB mice fed SD or KD (*N* = 8, 1way ANOVA with Sidak’s post test) normalized to wild type controls (*N* = 5, set to 1). **d** Quantification of myelination in the corticospinal tract from wild type mice, and Plp1tgB mice fed SD or KD (*N* = 7), showing g-ratio analysis as scatter plot (left panel) and the mean g ratio (right panel, 1way ANOVA with Tukey’s post test). **e** Relative frequency of sufficiently myelinated fibers (g ratio < 0.8), hypomyelinated fibers (g ratio > 0.8) or unmyelinated fibers (g-ratio = 1) in the CST of Plp1tgB fed SD or KD (*N* = 7, two-sided Student’s t-test of each group). **f** Rotarod analysis and **g** elevated beam test performance at 6 to 12 weeks of age (*N* = 7–8; 2way ANOVA with Sidak’s post test). Indicated are only significant differences between SD and KD fed Plp1tgB mice (*P < 0.05; **P < 0.01; ***P < 0.001). Scale bars 20 µm

