# Supplementary material for: Ketogenic diet ameliorates axonal defects and promotes myelination in Pelizaeus–Merzbacher disease
Source: Acta Neuropathol. 2019 Mar 27;138(1):147–61. doi: 10.1007/s00401-019-01985-2 (PMC6570703; doi:10.1007/s00401-019-01985-2)
Supplement: Supplementary file 1 — Supplementary material 1 (PDF 3826 kb) [file 401_2019_1985_MOESM1_ESM.pdf]

a

Features	PMD-A	PMD-B
Genetic mutation	hemizygous duplication Xq22	hemizygous duplication Xq22
Start of cholesterol	29 months	30 months
Dosage	200 mg/kg/d	125 - 590 mg/kg/d
Monitoring	Blood analyses[3], echocardiography, electrocardiogram; heart Doppler sonography, general physical, neurologic, neurophysiologic, neurodevelopmental, ophthalmologic, MRI	Blood analyses[3], carotid artery intima-media thickness, general physical, neurologic, neurophysiologic, neurodevelopmental, ophtalmologic, MRI
Q_{alb}	2.3 (28 months), 3.1 (34 months), normal age adapted range 0.5 - 4.0[1]	-
**Neurological status**		
Baseline	Severe generalized muscular hypotonia, tetraparesis, slight leg spasticity, sitting with support only, severe truncal and hand ataxia, slight nystagmus, alert, speaks few single words	Delayed psychomotor development: hand-motor function, cognitive and social development slightly below average; horizontal nystagmus, 1-word sentences, GMFCS 2 (walking with support)
After 1 year of suppl.	Slight improvement of muscular hypotonia, tetraparesis, slight leg spasticity, improved head control and hand function, supported sitting, ataxia slightly reduced, no nystagmus, very attentive, vivid expressions of wants and needs, nonverbal communication predominates	Positive psychomotor development (WPPSI III): average results in active and passive language performance; unsupported walking for some meters (GMFCS 1), ataxic, horizontal nystagmus
Current	Age 6.2 years: improved truncal stability and paresis, leg spasticity, walking with support, independent maneuvering of wheel chair, ataxia largely unchanged, attentive, good communication via talker	At 5 years: improved walking (300 m without support), improved cognitive and language abilities (55 months, full sentences in two languages), horizontal nystagmus

b

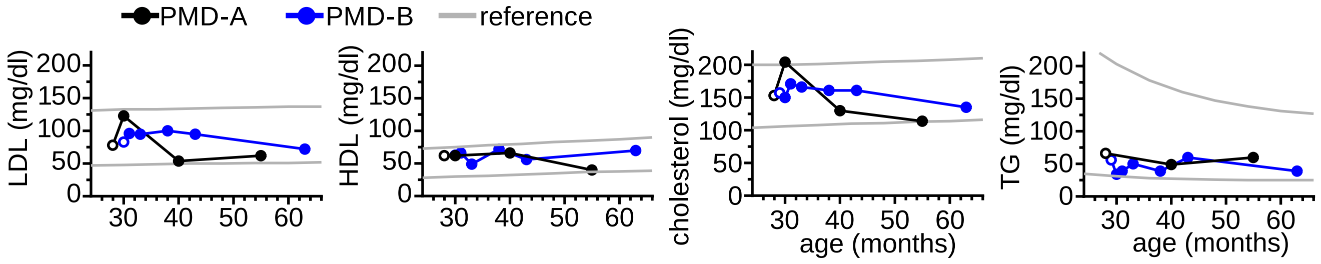

Supplemental Figure 1: Cholesterol supplementation in PMD patients.
(a) Study details with monitoring safety parameters, general developmental parameters, disease-specific neurological scores, as well as neurophysiologic and ophthalmologic assessments. Reiber analysis of Q_{alb} served to measure gross blood-brain barrier abnormalities. **(b)** Blood lipid values (open circle, baseline; filled circle, with cholesterol supplementation), grey lines delineate the age adapted P97 and P3 percentile reference values.

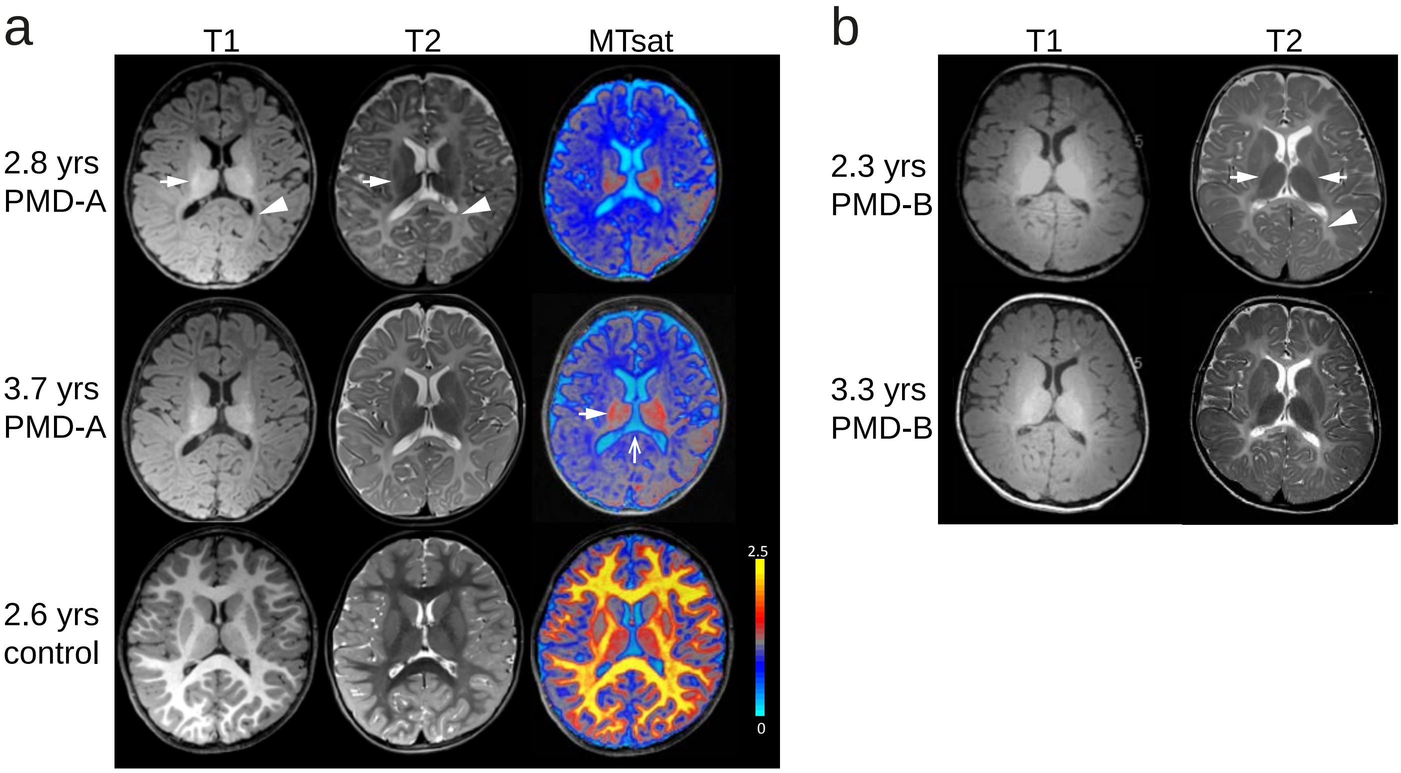

Supplemental Figure 2: Cholesterol supplementation in PMD patients - MRI study.
(a) MRI study of PMD patient A (PMD-A) who has received cholesterol beginning at 29 months of age and an age-matched infant control. Axial T1- (left) and T2-weighted (w) images (middle) were obtained prior to the begin of cholesterol supplementation (age 2.8 yrs) and after one year of cholesterol supplementation (age 3.7 yrs). MRI images demonstrate severe white matter hypomyelination with extensive signal hypointensities on T1-w and hyperintensities on T2-w images. We noted a minimally brighter signal on T1-w and corresponding lower signal on T2-w images within the internal capsule, e.g. in the posterior limb (arrow) and in the optic radiation (arrow head). This suggests a slight increase of myelin in these structures, as commonly observed in patients with *PLP1* duplication over the natural disease course [60, 67]. Signal intensities in white matter of the control infant are widespread, uniformly high on T1-w and low on T2-w MR-images representing myelination appropriate for that age. This control subject was diagnosed with neurological asymptomatic adrenoleukodystrohy. Right column shows semiquantitative magnetization transfer saturation (MTsat) maps overlayed onto corresponding T1-w images. The maps at both time points revealed pronounced and global myelin deficiency (blue and grey color in white matter, see color scale) compared to the control (yellow color in white matter). The small noticeable signal increase in the posterior limb of the internal capsule (filled arrow) and the splenium (open arrow) at 3.7 yrs compared to 2.8 yrs may reflect a discrete advancement of myelination as seen in T1-w and T2-w images.
(b) MRI study of PMD patient B (PMD-B) who has received cholesterol beginning at age 30 months. Axial T1-w and T2-w MRIs were acquired prior to the begin of cholesterol supplementation (age 2.3 yrs) and after one year of cholesterol supplementation (age 3.3 yrs). White matter displays widespread low (T1-w) and correspondingly high (T2-w) signal intensity reflecting pronounced hypomyelination. On T2-w MRIs, small symmetric spots with lower signal within the posterior limb of the internal capsule (arrow) and in optic radiation (arrow head) point to some myelin formation. However, myelination did not show major advancements and overall hypomyelination remained largely unchanged.

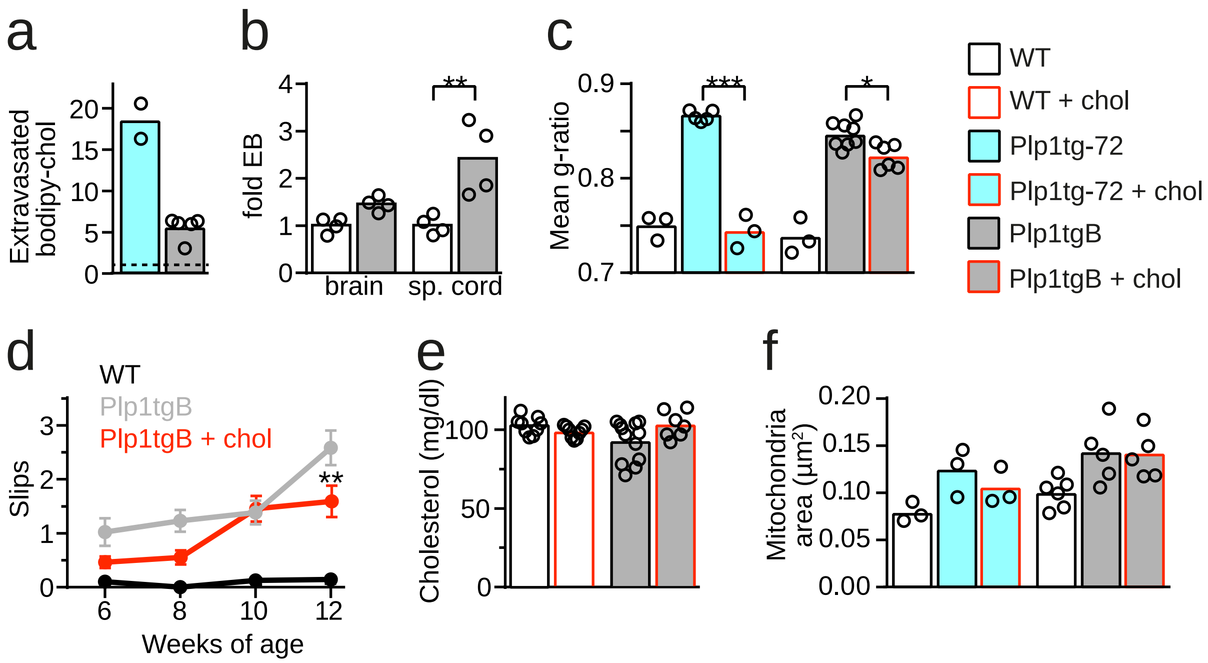

Supplemental Figure 3: Cholesterol treatment in Plp1tg-72 and Plp1tgB strains.
(a) Extravasation of bodipy-cholesterol in brain of Plp1tg-72 and Plp1tgB mutants normalized to wild type mice (set to 1, dashed line). **(b)** Extravasation of Evans Blue (EB) in brain and spinal cord of Plp1tgB mutants and WT controls (n=4 mice as indicated, 1way ANOVA with Tukey's post test). **(c)** Mean g-ratio as a measure of myelination show that in Plp1tgB mice, dietary cholesterol supplementation from 2-12 weeks of age only slightly ameliorated myelination in comparison to the strong effect in the original Plp1tg-72 strain (n=3-8 mice as indicated, 1way ANOVA with Tukey's post test of each treatment cohort). **(d)** Cholesterol supplementation slightly improved motor performance on a beam test in Plp1tgB mice (WT n=8; Plp1tgB n=15, Plp1tgB with cholesterol supplementation n=21), 2way ANOVA with Tukey's post test). **(e)** Total cholesterol in serum of WT and Plp1-tgB mice fed normal chow or cholesterol supplemented chow (n=7-12). **(f)** Quantification of mitochondrial area in cross sections of the corticospinal tract of Plp1tg-72 mice and Plp1tgB animals fed chow supplemented with or without cholesterol in comparison to wild type mice processed in parallel (n=3-6 mice, 1way ANOVA with Tukey's post test). Indicated are only significant differences between Plp1tg-72 and Plp1tgB treatment groups (* P<0.05, ** P<0.01, *** P<0.001). Subsequent experiments in this study were done with mutants of the Plp1tgB strain.

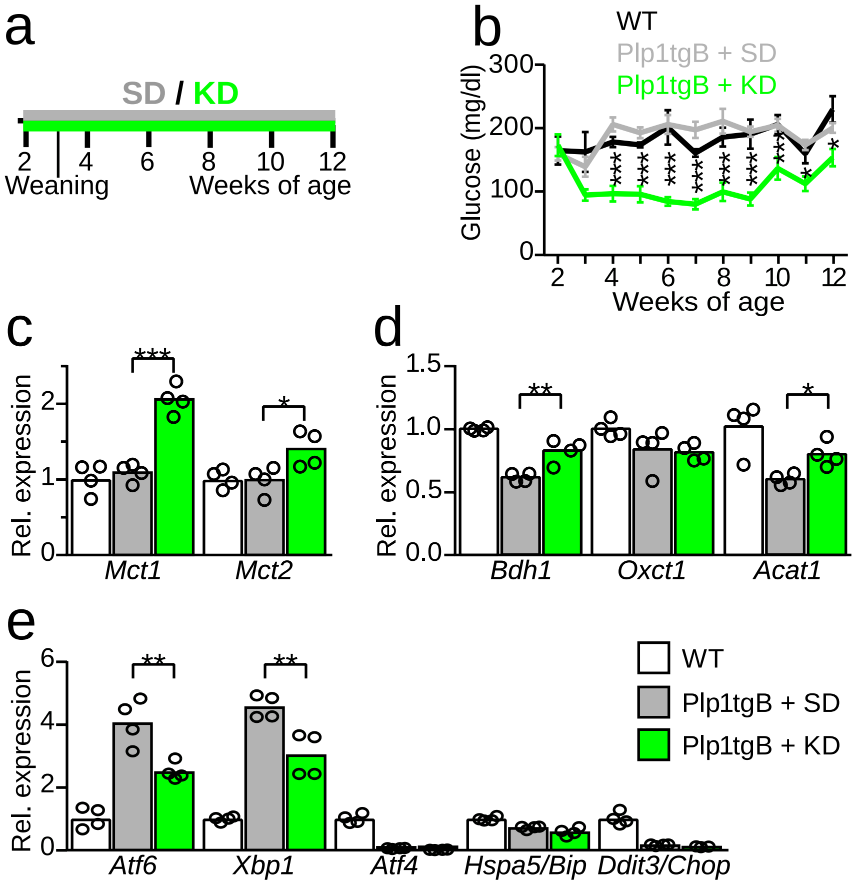

Supplemental Figure 4: Increased ketone body uptake and ketolysis in KD fed animals. **(a)** Treatment paradigm, Plp1tgB mice were fed SD or KD from 2 to 12 weeks of age. **(b)** Physiological parameters changed as expected, decreasing mean blood glucose ± SEM (n=4 (WT), n=8(Plp1tgB fed SD), n=9 (Plp1tgB fed KD), 2way ANOVA with Tukey's post test). **(c-e)** Quantitative RT-PCR on dissected lumbar spinal cord tissue determining (c) expression of monocarboxylate transporters for ketone body uptake *Mct1* and *Mct2* and (d) for ketolysis *Bdh1* (3-hydroxybutyrate dehydrogenase 1), *Oxct1* (3-oxoacid CoA transferase 1) and *Acat1* (acetyl-Coenzyme A acetyltransferase 1), or (e) markers for ER stress reponse *Atf6* (activating transcription factor 6), *Xbp1* (X-box binding protein 1), *Atf4*, *Hspa5* (heat shock protein 5) also termed *Bip* or *Grp78*, and *Ddit3* (DNA-damage inducible transcript 3) also termed *Chop* (C/EBP homoologous protein). Significance was tested using 1way ANOVA with Tukey's multiple comparison test, indicated are only significant differences between SD and KD fed Plp1tgB mice (n=4 animals, * P<0.05, ** P<0.01, *** P<0.001).

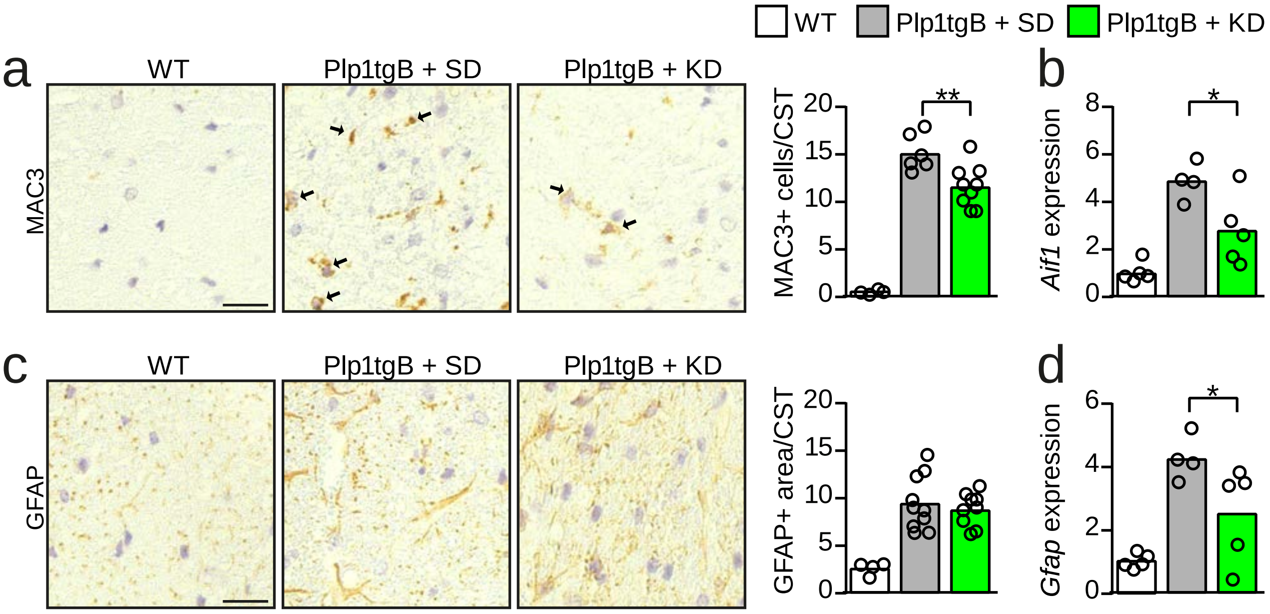

Supplemental Figure 5: Ketogenic diet ameliorates gliosis in Plp1tgB animals.
(a) MAC3 staining in the corticospinal tract of wild type and Plp1tgB mice fed SD or KD with quantification on the right, showing the mean with individual values (n=4-9 mice). **(b)** *Aif1* expression by quantitative RT-PCR on spinal cord normalized to wild type animals (n=4-5). **(c)** GFAP staining with quantification of astrogliosis (GFAP positive area, n=4-10). **(d)** *Gfap* expression by quantitative RT-PCR on spinal cord normalized to wild type animals (n=4-5). Significance was evaluated by 1way ANOVA with Tukey‘s post test. Indicated are only significant differences between SD and KD fed Plp1tgB mice (* P<0.05, ** P<0.01). Scale bars, 20 µm.

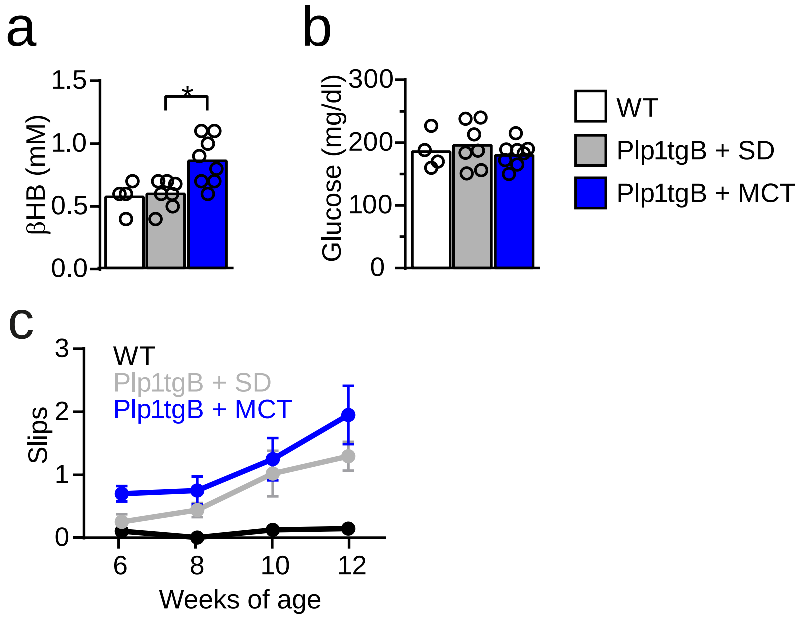

Supplemental Figure 6: Medium-chain triglyceride ketogenic diet is not effective in Plp1tgB animals.
Plp1tgB mice were fed medium-chain triglyceride diet (MCT) or SD from 2-12 weeks of age and mean blood values of **(a)** beta-hydroxybutyrate (ßHB) and **(b)** glucose were determined at the end of the treatment period (n=4-8 animals, 1way ANOVA with Tukey's post test). Indicated are only significant differences between SD and MCT fed Plp1tgB mice (* P<0.05). **(c)** Beam test to evaluate motor performance from 6 to 12 weeks of age (n=5 animals).

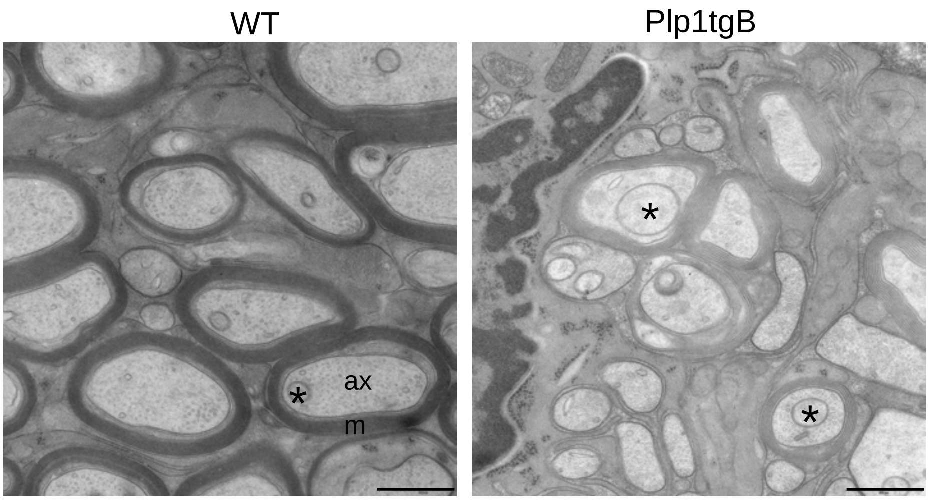

Supplemental Figure 7: Plp1tgB mice develop mitochondrial defects before demyelination. High pressure frozen processed optic nerve of 3 weeks old Plp1tgB and WT mice show enlarged mitochondria profiles in myelinated and unmyelinated axons. Scale bars, 500 nm (*, mitochondria; ax, axon; m, myelin).

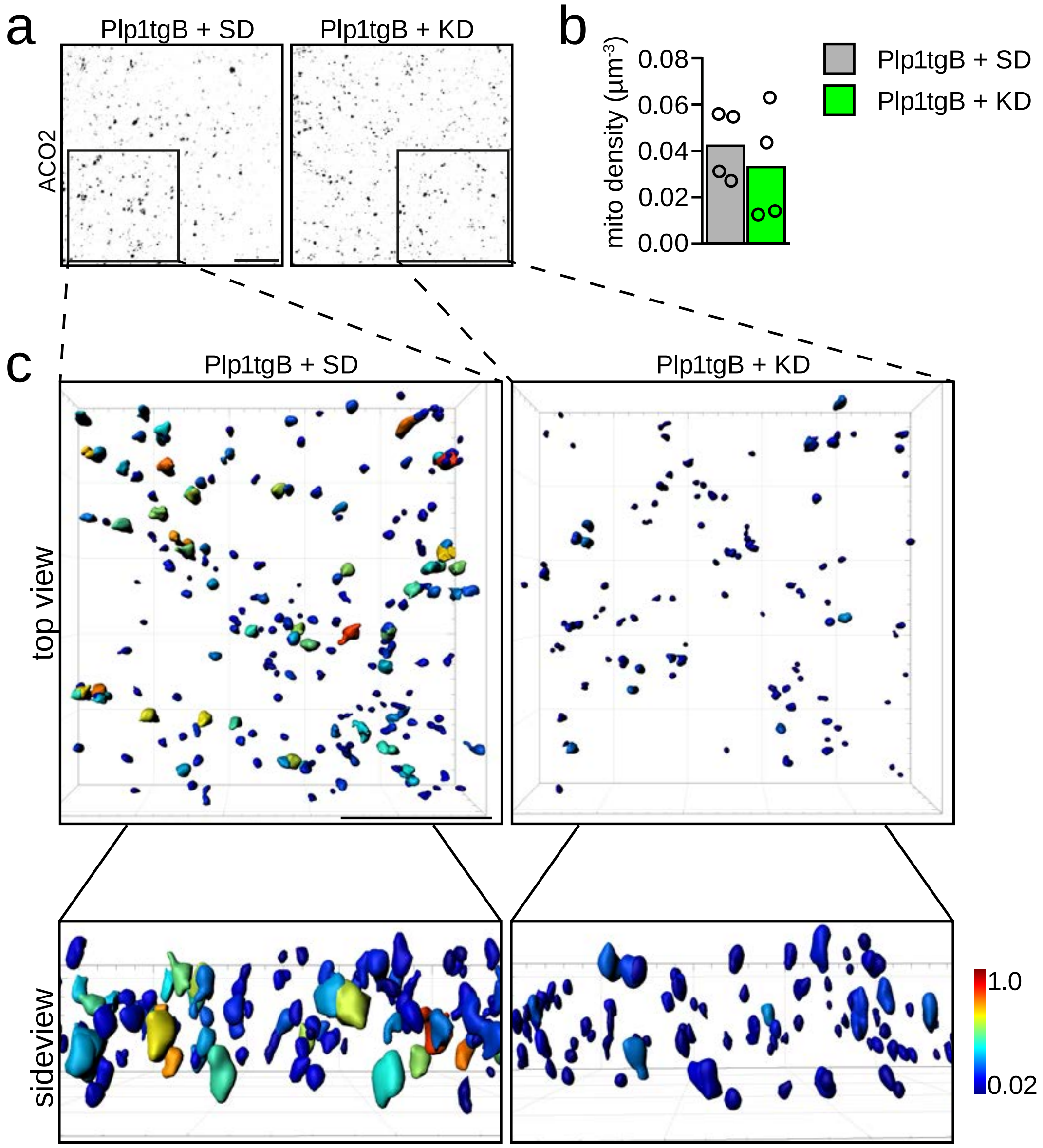

Supplemental Figure 8: Altered mitochondrial sizes in Plp1tgB mice but unchanged density
(a) Inverted picture of a maximum intensity projection of ACO2 immunolabeled spinal cord sections from Plp1tgB mice fed SD or KD with **(b)** quantification of mitochondrial density. **(c)** 3D modelled mitochondrial profiles from deconvolved confocal image stacks were color coded according to volume (0.02-1.0 μm^3). Scales, 10 µm.

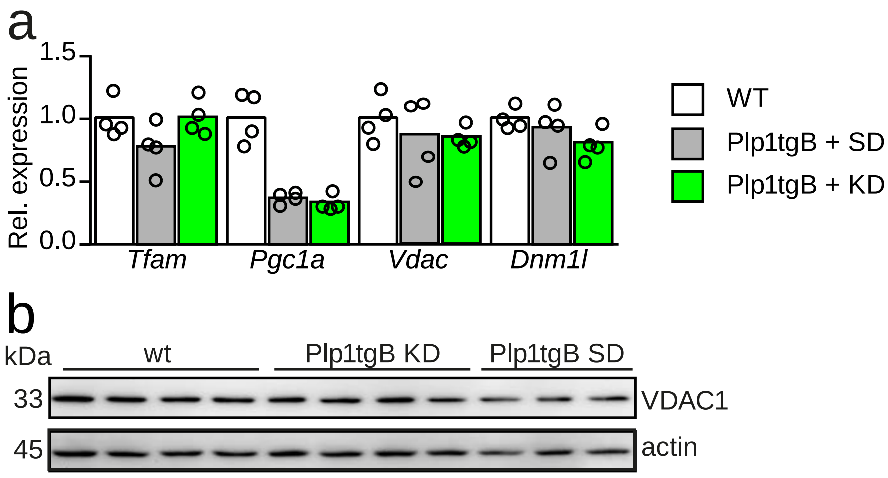

Supplemental Figure 9: KD does not alter expression of mitochondrial markers. **(a)** Quantitative RT-PCR determining the expression of mitochondrial markers Tfam, Pgc1a, Vdac, and Dmn1l in spinal cord shows comparable levels in Plp1tgB mice fed SD and KD compared to wild type animals (n=4). **(b)** Western Blot detecting VDAC1 in spinal cord of wild type mice (n=4) and Plp1tgB mice fed SD (n=3) or KD (n=4). Equal protein loading was confirmed by staining of actin.

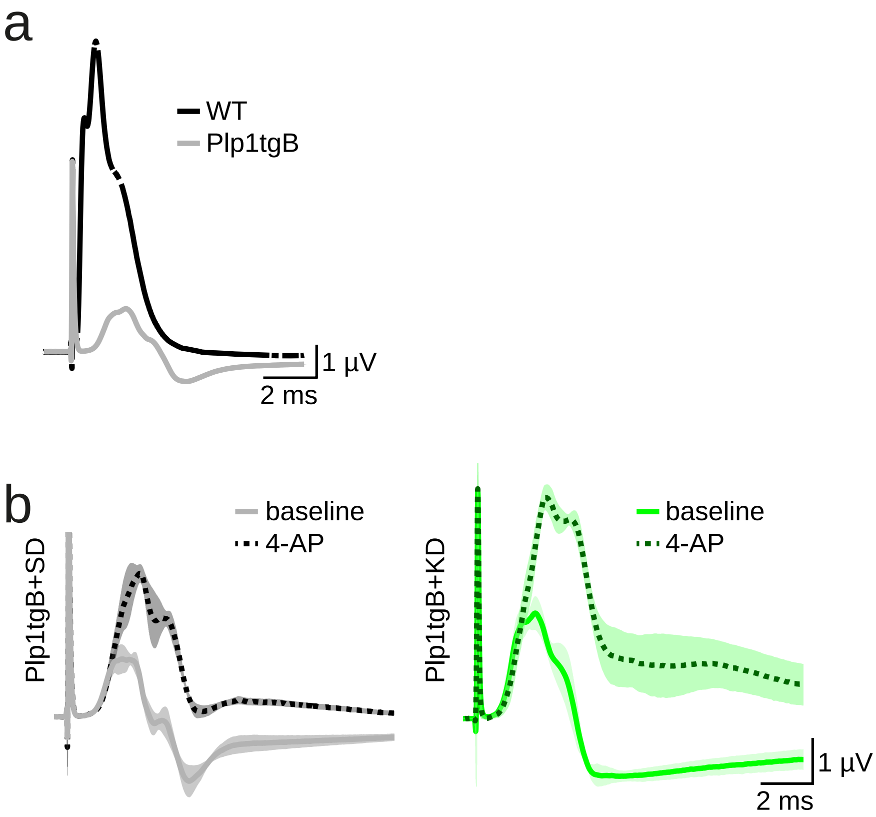

Supplemental Figure 10: After-hyperpolarization is sensitive to potassium channel blockage.
(a) CAP recording of optic nerves from SD fed Plp1tgB mice (n=11) in comparison to a typical WT trace at baseline conditions. **(b)** CAP recordings of optic nerves from Plp1tgB mice fed SD (n=4) or KD (n=3) at baseline conditions with or without bath application of the voltage gated potassium channel blocker 4-aminopyridine (4-AP, 50 µM). Data is shown as mean ± SEM.

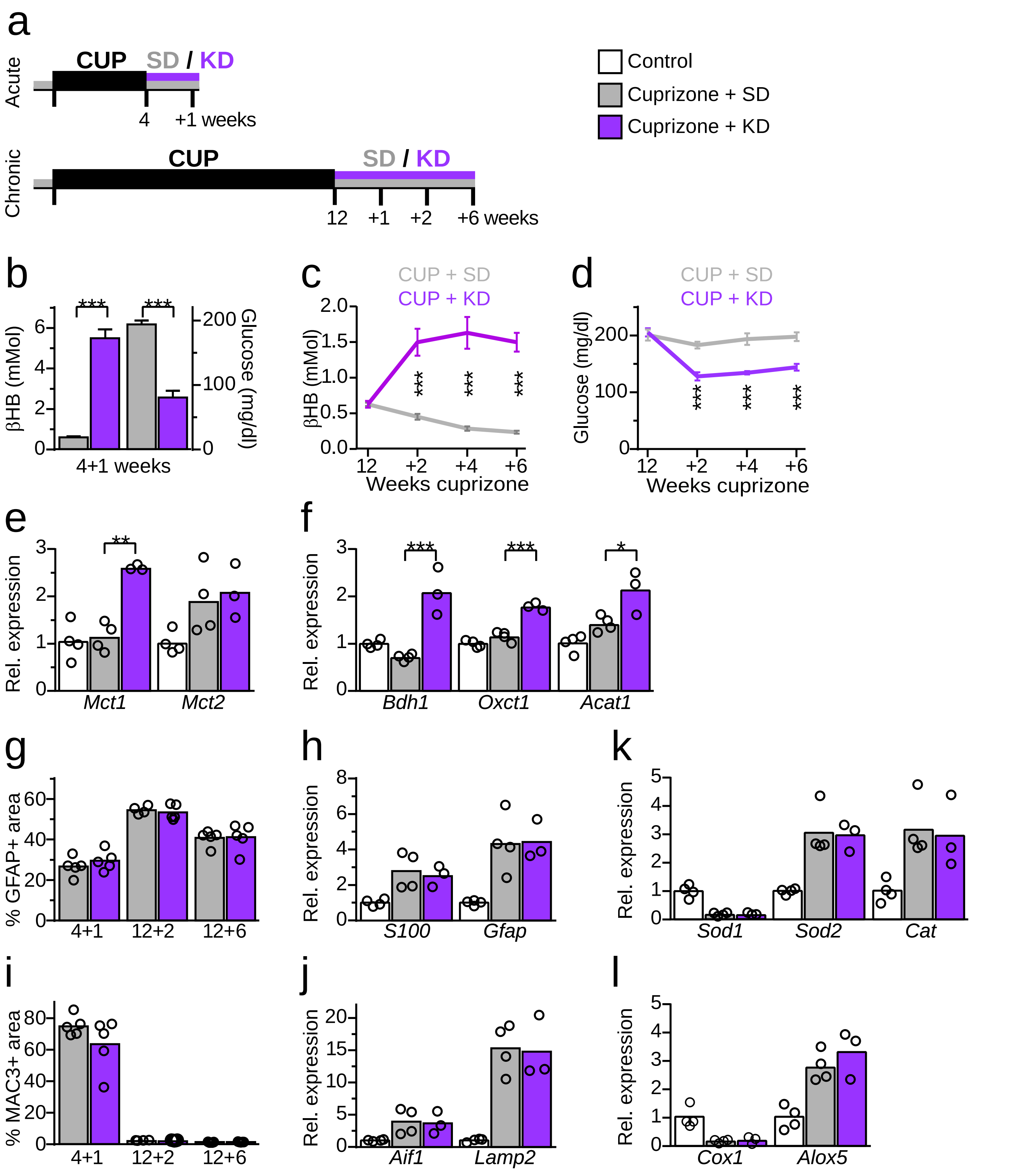

Supplemental Figure 11: Feeding KD to mice after cuprizone mediated demyelination.
(a) Treatment paradigms. In an acute paradigm, cuprizone (CUP) in SD was fed for 4 weeks, followed by feeding SD or KD for one week. In the chronic paradigm, cuprizone was fed for 12 weeks, followed by KD or SD for up to 6 weeks. **(b-d)** Mean blood levels of beta-hydroxybutyrate (βHB) and glucose (n=5 animals). Significance was evaluated by Student‘s t-test (b) or 2way ANOVA with Bonferronis‘s post test (c-d). **(e-f)** Relative gene expression in dissected corpus callosum of mice after 12 weeks cuprizone, followed by 2 weeks KD or SD (12+2, n=3-4), normalized to untreated controls (set to 1, n=4). Quantified were (e) monocarboxylate transporters *Mct1* and *Mct2*, and (f) the enzymes essential for ketone body utilization *Bdh1, Oxct1, Acat1*. **(g-h)** Astrogliosis measured (g) histochemically as percent GFAP positive area in the corpus callosum and (h) by quantitative RT-PCR of the astroglial genes *Gfap* and *S100*. **(i-j)** Microgliosis measured (i) histochemically as percent MAC3 positive area in the corpus callosum and (j) by quantitative RT-PCR of the microglial genes *Aif1* and *Lamp2*. (1way ANOVA with Tukey’s post test). **(k, l)** Relative gene expression in dissected corpus callosum of mice after 12 weeks cuprizone, followed by 2 weeks KD or SD (12+2, n=3-4), normalized to untreated controls (set to 1, n=4). Quantified were (k) enzymes for detoxification of reactive oxygen species / peroxides superoxide dismutases (*Sod1, Sod2*) and catalase (*Cat*) and (l) enzymes involved in the synthesis of proinflammatory eicosanoids, i.e. cyclooxygenases (*Cox1, Cox2*) and arachidonate 5-lipoxygenase (*Alox5*). Indicated are only significant differences between cuprizone groups (* P<0.05, ** P<0.01, *** P<0.001).

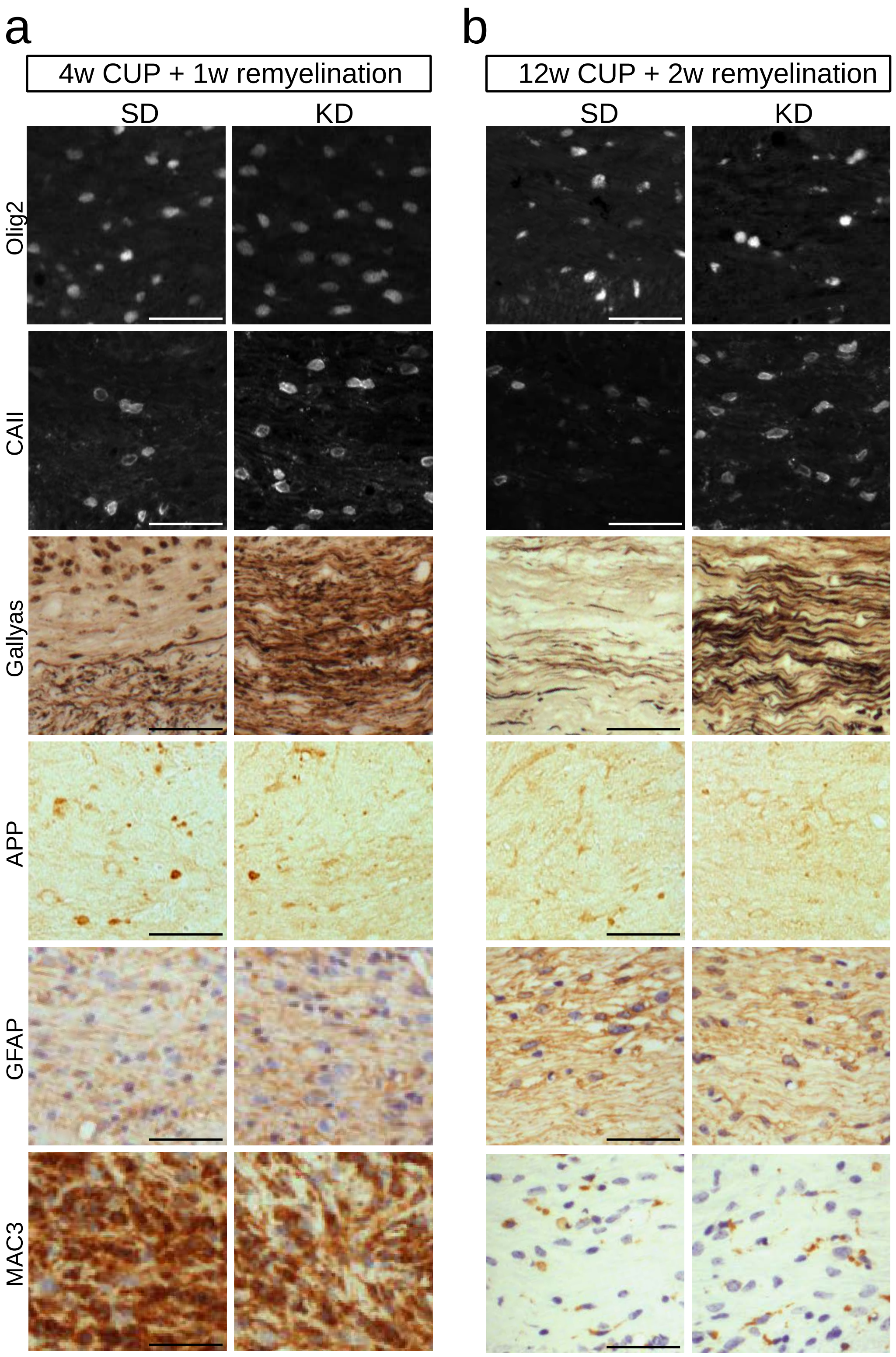

Supplemental Figure 12: Histochemical evaluation of ketogenic diet fed animals during remyelination. Representative pictures of corpus callosum sections stained for oligodendroglia (Olig2), mature oligodendrocytes (CAII), myelin (Gallyas), axonal spheroids (APP), astrogliosis (GFAP), and microgliosis (MAC3) from mice **(a)** after 4 weeks demyelination with cuprizone followed by 1 week remyelination with SD or KD or **(b)** after 12 weeks demyelination followed by 2 weeks remyelination with SD or KD. Scale bars, 50 µm.

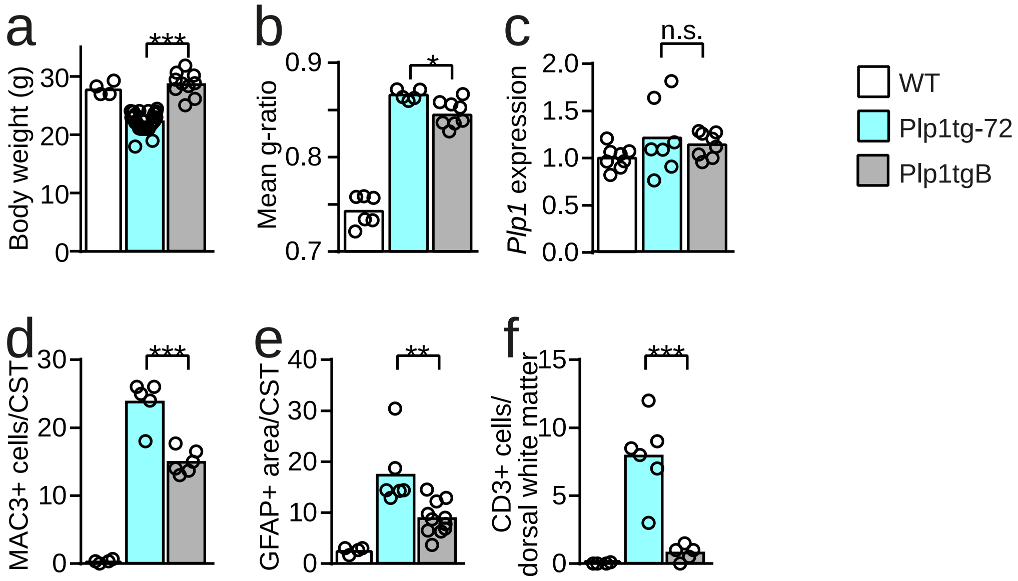

Supplemental Figure 13: Characterization of Plp1tg-72 and Plp1tgB strains.
(a) Body weight of WT, Plp1tg-72 and Plp1tgB mice at 12 weeks of age (n=4-26 mice as indicated). **(b)** Mean g-ratio in spinal cord CST of WT, Plp1tg-72 and Plp1tgB mice (n=5-8 mice). **(c)** *Plp1* expression by RT-qPCR in spinal cord of WT, Plp1tg-72 and Plp1tgB mice (n=7-8 animals per strain). **(d)** Mean MAC3 positive cells in the corticospinal tract of WT, Plp1tg-72 and Plp1tgB mice (n=4-6 mice). **(e)** Mean GFAP positive area in the corticospinal tract of WT, Plp1tg-72 and Plp1tgB mice (n=4-11 mice). **(f)** Mean CD3 positive cells in the dorsal white matter of WT, Plp1tg-72 and Plp1tgB mice (n=4-6 mice). Indicated are only differences between Plp1tg-72 and Plp1tgB groups (* P<0.05, ** P<0.01, *** P<0.001; 1way ANOVA with Tukey's post test).
