# Supplementary material for: Ketogenic diet ameliorates axonal defects and promotes myelination in Pelizaeus–Merzbacher disease
Source: Acta Neuropathol. 2019 Mar 27;138(1):147–61. doi: 10.1007/s00401-019-01985-2 (PMC6570703; doi:10.1007/s00401-019-01985-2)
Supplement: Supplementary file 2 — Supplementary material 2 (DOCX 18 kb) [file 401_2019_1985_MOESM2_ESM.docx]

### Supplemental table 1: Primer sequences

Primers used for expression analysis were intron-spanning (5’-3’):

| *Hprt1* | for:  rev: | TCCTCCTCAGACCGCTTTT  CCTGGTTCATCATCGCTAATC |
| --- | --- | --- |
| *Rplp0* | for:  rev: | GATGCCCAGGGAAGACAG  ACAATGAAGCATTTTGGATAATCA |
| *Plp1* | for:  rev: | TCAGTCTATTGCCTTCCCTAGC  AGCATTCCATGGGAGAACAC |
| *Olig2* | for:  rev: | AGACCGAGCCAACACCAG  AAGCTCTCGAATGATCCTTCTTT |
| *Car2* | for:  rev: | CAAGCACAACGGACCAGA  ATGAGCAGAGGCTGTAGG |
| *Gfap* | for:  rev: | TGCTCCTGCTTCGAGTCCTT  CAAGAGGAACATCGTGGTAAAGA |
| *S100* | for:  rev: | AACAACGAGCTCTCTCACTTCC  CTCCATCACTTTGTCCACCA |
| *Aif-1 (*IBA1*)* | for:  rev: | TGTTTTTCTCCTCATACATCAGAATC  CCGAGGAGACGTTCAGCTAC |
| *Lamp2* | for:  rev: | AAGGTGCAACCTTTTAATGTGAC  TGTCATCATCCAGCGAACAC |
| *Slc16a1 (*MCT1*)* | for:  rev: | ATGCTGCCCTGTCCTCCT  CCACAAGCCCAGTACGTGTAT |
| *Slc16a7 (*MCT2*)* | for:  rev: | TCGTGGAGTGTTGTCCAGTT  TCCAGTTATATCAAGCAATTTACCA |
| *Acat1* | for:  rev: | AGGGAAGTTTGCCAGTGAGA  TTCACCACCACATCTGGTTTAC |
| *Bdh1* | for:  rev: | GAGCTACGGGTTCAGACGAG  TGGCACCAAGTTGTAAGACG |
| *Oxct1* | for:  rev: | GGCCAACTGGATGATACCTG  GGAACTGGACACCAAATCCA |
| *Atf6* | for:  rev: | GACAGCTCTTCGCTTTGGAC GGACGAGGTGGTGTCAGAG |
| *Xbp1* | for:  rev: | AGGAGTTAAGAACACGCTTGGG GGTCCAACTTGTCCAGAATGC |
| *Atf4* | for:  rev: | ATGATGGCTTGGCCAGTG TCTCCAACATCCAATCTGTCC |
| *Hspa5 (*BIP*)* | for:  rev: | CTGAGGCGTATTTGGGAAAG CAGCATCTTTGGTTGCTTGTC |
| *Ddit3 (*CHOP*)* | for:  rev: | GCGACAGAGCCAGAATAACA GATGCACTTCCTTCTGGAACA |
| *Tfam* | for:  rev: | CAAAGGATGATTCGGCTCAG  AAGCTGAATATATGCCTGCTTTTC |
| ***Ppargc1a*** | for:  rev: | GAAAGGGCCAAACAGAGAGA  GTAAATCACACGGCGCTCTT |
| *Vdac* | for:  rev: | ACCTTTGATTCGTCATTCTCG  TGCTCCCTCTTGTACCCTGT |
| *Dmn1l* | for:  rev: | CTGGATCACGGGACAAGG  GTTGCCTGTTGTTGGTTCCT |
| *Alox5* | for:  rev: | AGCTGTAAACTTCGGCCAGT  GATGGTGACCACACCCTTG |
| *Cat* | for:  rev: | AGCGGATTCCTGAGAGAGTG  ATCGTGGGTGACCTCAAAGT |
| *Cox1 (Ptgs1)* | for:  rev: | CCACTCGCCTCATCCTTAGA  GGAGCTCCGGGTCAAACT |
| *Sod1* | for:  rev: | CCATCAGTATGGGGACAATACA  ATGGACACATTGGCCACAC |
| *Sod2* | for:  rev: | TGCTCTAATCAGGACCCATTG  GTAGTAAGCGTGCTCCCACAC |
